# Longitudinal basophil activation testing for assessing response to low-dose initiation, slow-escalation peanut oral immunotherapy in patients with high Ara h 2-sIgE

**DOI:** 10.3389/falgy.2026.1902025

**Published:** 2026-10-07

**Authors:** Jinguo Chen, J. Joanna Yu, Jing Yu, Lili Wan, Joseph Chiao, Athena Economides, Dingfen Han, H. Henry Li

**Affiliations:** 1 Virant Diagnostics Laboratory, Wheaton, MD, United States; 2 Institute for Asthma and Allergy (IAA), Chevy Chase, MD, United States; 3 Department of Medicine, School of Medicine, Johns Hopkins University, Baltimore, MD, United States

**Keywords:** Ara h 2-specific IgE, basophil activation test, biomarker, desensitization, oral immunotherapy, peanut allergy, specific IgG4

## Abstract

**Background:**

Peanut oral immunotherapy (pOIT) can achieve desensitization, yet patients with high peanut-sIgE may experience more adverse reactions and treatment failure. We evaluated a conservative pOIT protocol in children with high Ara h 2-sIgE (≥40 kU/L) and examined longitudinal basophil activation test (BAT) and sIgE/sIgG4 trajectories as candidate biomarkers.

**Methods:**

Thirty-two peanut-allergic children underwent pOIT, including 16 with high and 16 with lower Ara h 2-sIgE. All received low-dose initiation and slow up-dosing. Clinical outcomes, safety, and immunologic markers were evaluated longitudinally for up to 36 months.

**Results:**

Effective desensitization (≥300 mg peanut protein) was achieved in 68.8% of high-sIgE and 62.5% of lower-sIgE participants. One severe adverse event occurred in each subgroup, with no treatment-related discontinuations. Peanut and Ara h 2/6-sIgE levels rose transiently before gradually declining, whereas sIgG4 increased early, leading to a reduced sIgE/sIgG4 ratio. A ratio <50 was associated with effective desensitization. BAT responses initially increased and subsequently declined, with progressive increase in the activation threshold. Baseline BAT and sIgE were not associated with desensitization. However, BAT area under the curve (AUC) <100 was significantly associated with effective desensitization with ROC AUC of 0.784 (bootstrap 95% CI 0.536–0.983). Exploratory time-lagged analyses did not establish prospective predictive utility.

**Conclusions:**

Low-dose initiation, slow-escalation pOIT achieved effective desensitization in patients with high Ara h 2-sIgE, without treatment-related discontinuations. Longitudinal reductions in BAT and sIgE/sIgG4 ratio were associated with increased ingested dose. These exploratory biomarkers may provide adjunctive information during pOIT, but prospective validation is required.

## Introduction

Peanut allergy has risen markedly worldwide over the past decade, affecting approximately 2%–3% of children in Western countries (1). Unlike milk or egg allergy, which often resolves in childhood (2), peanut allergy is frequently lifelong, with only 10%–20% of affected children achieving natural resolution (3, 4). Consequently, strict dietary avoidance remains the standard of care but is associated with frequent accidental exposures and reduced quality of life (QoL).

Allergen immunotherapy (AIT) provides an active approach to desensitization and is increasingly adopted in clinical practice globally (4). The 2020 FDA approval of Palforzia® (formerly AR101) marked an important milestone in standardizing peanut oral immunotherapy (pOIT) (4–6). Meta-analyses of clinical trials demonstrate desensitization (DS) in roughly two-thirds of patients (7) and sustained unresponsiveness (SU) in selected populations, particularly younger children (8, 9). Nevertheless, SU is not equivalent to cure (3), and protection often wanes over time without continued ingestion with approximately 50% loss of tolerance every three months (10).

While achieving SU or long-term tolerance remains a central goal in efficacy assessment (11), for many families and patients, protection against accidental exposures is their primary clinical objective. In real-world settings, most unintended peanut exposures involve small amounts of peanut, typically well below 100 mg, approximately the population ED50 (eliciting dose). A reactivity threshold of ≥300 mg is estimated to protect against >95% of accidental exposures and represents a clinically meaningful benchmark (12). Achieving and maintaining this threshold can substantially improve QoL, even when ongoing peanut ingestion remains necessary (13).

Currently, no reliable biomarker exists to predict or monitor adverse reaction (AR) risk or guide patient selection for AIT. Baseline allergen-specific IgE (sIgE) levels >50 kU/L are often associated with increased risk of anaphylaxis and OIT failure (14–16), although findings are inconsistent across studies. Ara h 2 is the most informative serologic marker of peanut allergy severity (17), yet its value for predicting OIT outcome remains unknown. For high-risk patients (e.g., sIgE >50 kU/L), modified approache—including slow dose increment, low maintenance dosing or sublingual immunotherapy (SLIT)—have been proposed to enhance safety while preserving efficacy (14, 18).

Despite advances in understanding the immunological mechanisms of OIT (19) and protocol optimization, validated biomarkers for monitoring desensitization and guiding individualized therapy remain limited. Double-blind, placebo-controlled food challenges (DBPCFC), while the gold standard, are resource-intensive and carry procedural risk. Candidate biomarkers include humoral, cellular, and metabolic measures. A hallmark of successful AIT is the induction of blocking IgG antibodies, particularly IgG1 and IgG4, which may increase 10 to 100-fold from baseline (20). However, the relationship between IgG4 induction and clinical outcomes remains inconsistent (21). Conventional serologic markers (sIgE, sIgG4) reflect humoral responses but do not directly capture effector-cell reactivity. In contrast, the basophil activation test (BAT), which quantifies allergen-induced CD63 expression via FcεRI signaling, provides a functional readout of effector-cell responsiveness and has shown promise in assessing OIT outcomes (22, 23).

In this retrospective, real-world study, we evaluated whether peanut-allergic patients with high Ara h 2–specific IgE could safely achieve clinically meaningful desensitization using a low-dose initiation, slow-escalation OIT protocol. We further examined longitudinal changes in BAT and sIgE/sIgG4 ratios as dynamic candidate biomarkers of immune modulation and treatment response.

## Methods

### Study design and population

This retrospective, real-world study was conducted at a single allergy clinic center between September 2021 and June 2025. Thirty-two patients aged 4–18 years with physician-diagnosed peanut allergy who underwent standard-of-care peanut OIT (pOIT) were included in this study (patient flow diagram seen in Supplementary Figure S1). Inclusion criteria comprised a history of clinical reactivity, a positive skin prick test (SPT; wheal ≥3 mm), peanut-specific IgE (sIgE) level >0.35 kU/L, and at least two basophil activation test (BAT) assessments performed ≥6 months apart. Patients with uncontrolled asthma or concurrent milk allergy, which could confound BAT results (24), were excluded. All eligible patients meeting the prespecified inclusion criteria during the study period were included; no *a priori* sample-size calculation was performed. Patients with incomplete OIT treatment were excluded from this biomarker analytic cohort study.

Participants were stratified *a priori* by baseline Ara h 2-sIgE using a threshold of 40 kU/L, selected based on published clinical data identifying approximately 40 kU/L as a level associated with a high likelihood of clinically relevant peanut allergy (25). This threshold was used to define a subgroup with marked sensitization and was not intended as an OIT-response or therapeutic cutoff. Sixteen patients with Ara h 2-sIgE ≥40 kU/L formed the “high sIgE” subgroup, while the remaining 16 with lower values constituted the “lower sIgE” comparison subgroup. All participants followed the same OIT protocol. De-identified data were analyzed under approval from the Salus Institutional Review Board (IRB).

### Peanut formulation and OIT protocol

Consistent with most real-world practice, OIT was performed using food-based formulations rather than a standardized pharmaceutical product (3). In this study, peanut protein doses were prepared from in-house mixed nut butter (MNB) and peanut butter (PB), or commercially whole peanuts, peanut butter-containing candy (e.g., Reese's Pieces, M&M's), PB2 powder, with standardized peanut protein content (Supplementary Table S1). Before reaching an SCD of 300 mg, patients received peanut doses prepared from in-house MNB or PB to ensure accurate dose administration. After attaining an SCD >300 mg, patients gradually transitioned to whole peanuts or commercially available peanut-containing products.

To minimize risk, oral food challenges (OFCs) were omitted for most patients. The protocol employed a very low initial dose, slow dose escalation, and a prolonged up-dosing phase lasting 1–2 years (Supplementary Table S2). Briefly, the planned schedule comprised 18 dose levels beginning at 0.1 mg and progressing to 364.4 mg by Visit 18, thereby crossing the clinically meaningful 300 mg threshold (Figure 1A), a threshold estimated to protect against >95% of accidental exposures and considered a clinically meaningful level of “real-world” protection (4, 12), even in patients who remain allergic (9). Dose escalations were typically performed every 14–28 days during clinical visits, provided prior doses were well tolerated or associated only with mild symptoms. The maintenance dose was set at the highest individually tolerated dose, with continued daily ingestion to sustain desensitization. Given that long-term clinical benefit from AIT may require >3 years of treatment (21), participants continue daily intake beyond 36 months; however, data beyond this time point were not included in the present analysis. Real-world adherence, including missed doses, and dosing-related reactions, was systematically documented.

**Figure 1:**
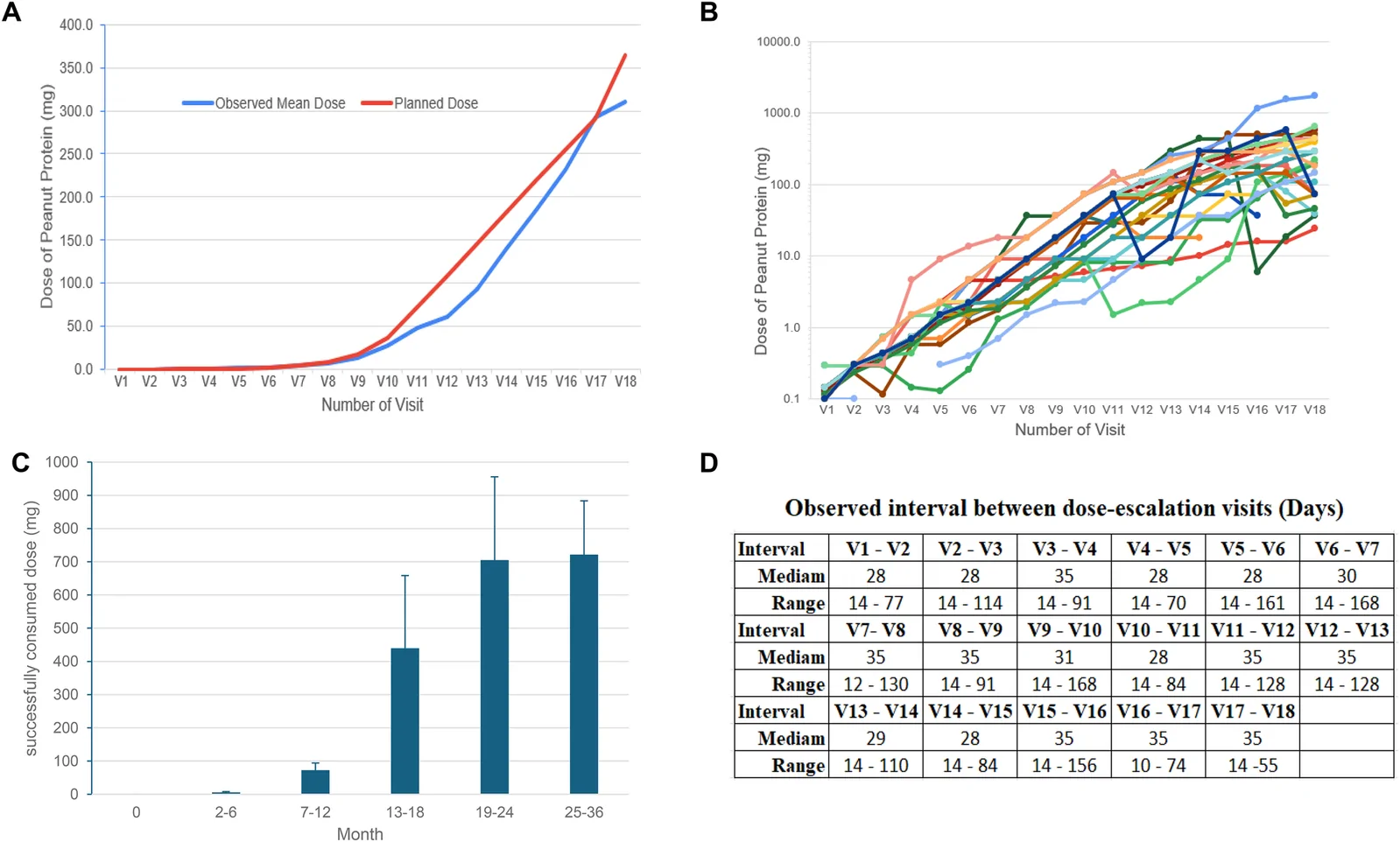
Peanut oral immunotherapy (pOIT) protocol with low-dose initiation and slow up-dosing using an office-based peanut product. **(A)** Planned pOIT regimen initiated at 0.1 mg of peanut protein, followed by slow, prolonged up-dosing targeting an ingested dose exceeding the clinically meaningful 300 mg threshold by visit 18. The observed mean ingested dose closely paralleled the planned dose trajectory. **(B)** All 32 participants completed the first 18 clinic visits over 12–24 months. Actual ingested doses varied among participants, and some scheduled doses were missed. After reaching the target desensitization threshold, some participants continued dose escalation to increase the tolerated daily dose and maintain desensitization (data not shown). **(C)** Highest SCD (successfully consumed dose) during each treatment period, expressed as mean ± SEM. **(D)** Although the planned interval between dose-escalation visits was 14−28 days during the first 18 visits, the observed intervals were generally longer than planned and longer than the 14-day interval recommended for Palforzia®, reflecting an extended-interval, prolonged up-dosing protocol.

### Clinical assessments

The clinical effectiveness of pOIT was evaluated with desensitization. Successfully consumed dose (SCD) was defined as the total daily peanut-protein dose that was successfully consumed. Effective desensitization was defined as achieving an SCD of ≥300 mg peanut protein. The primary clinical endpoint is attainment of a successfully consumed daily peanut-protein dose (SCD) ≥300 mg during OIT. Safety was assessed by documenting the frequency and severity of dose-limiting symptoms during OIT. Symptom severity was graded according to World Allergy Organization (WAO) and Consortium for Food Allergy Research (CoFAR) Criteria, Version 3.0 (26, 27); only moderate-to-severe reactions were considered dose-limiting. Mild symptoms could prompt temporary withholding of escalation or continuation at the same dose according to clinical judgment but were not classified as protocol-defined dose-limiting events (Supplementary: Methods). Anaphylaxis was defined per the 2020 WAO updated grading system.

### Basophil activation test (BAT)

BAT was modified from a flow cytometry-based protocol (28). Briefly, within 24 h of blood collection, 200 µL of heparinized whole blood was incubated for 20 min at 37 °C with PBS or KLH (negative controls), anti-IgE antibody or fMLP (positive controls), or five serial concentrations of peanut protein (10,000, 1,000, 100, 10 and 1 ng/mL). Cells were subsequently stained with fluorescent antibodies against CD63 and CD123/CD193, incubated for 30 min at 2–8° C, lysed (BD lysing solution), washed with PBS, and acquired on an Attune NxT flow cytometer (Thermo Fisher). Data were analyzed with FCS Express 7 (*de novo* Software). Basophils were gated as CD123+/CD193+ with low side scatter. A minimum of 500 basophils per sample was analyzed, and CD63 expression was quantified. Gating was standardized using PBS controls (2 ± 0.25% CD63+). Basophil activation ≥5% CD63⁺ cells was considered positive.

Dose–response curves were generated by plotting the percentage of CD63⁺ basophils against the peanut protein concentrations. Given the heterogeneous and often bell-shaped nature of BAT curves (24), the area under the curve (AUC) was used as the primary integrated measure. BAT AUC was calculated by trapezoidal integration of %CD63+ basophils across the five log10-transformed peanut concentrations. The maximum dose response (MDR) was also recorded for clinical comparison.

BAT was validated and is in clinical use as a laboratory-developed test (LDT) conducted at our CLIA-certified, CAP-accredited Virant Laboratory. The core analytical validation data are summarized in the Supplementary Methods (Supplementary Tables S3–S5). BAT assays were performed, analyzed, and initially reported by one investigator who did not have access to immunoglobulin results or clinical outcome data at the time of interpretation, and the BAT results were subsequently audited by a second investigator under the same blinding conditions. Clinical treatment and outcome data were collected separately by another investigator without prior knowledge of laboratory results. The laboratory and clinical datasets were subsequently integrated for statistical analysis by another author.

### Total IgE, sIgE and sIgG4 measurements

Serum levels of total IgE, peanut- specific and two component-specific IgE (Ara h 2, Ara h 6), and corresponding sIgG4 were measured at baseline, during up-dosing phase, and during maintenance. Assays were performed using the ImmunoCAP platform (Phadia Immunoassay Analyzers; Thermo Fisher Scientific Inc., Waltham, MA) at a CLIA-certified commercial laboratory. For graphical presentation and descriptive summaries, values exceeding the upper limit of quantification (100 kU/L for sIgE, 30 mg/L for sIgG4) were displayed at the assay upper limit. For inferential longitudinal analyses, these observations were treated as right-censored.

### Statistical analysis

Continuous variables are presented as mean ± standard error of the mean (SEM). BAT AUC and SCD were analyzed using longitudinal mixed-effects models with participant-specific random intercepts and assessment period as a categorical fixed effect. Skewed continuous outcomes were analyzed on the natural-log scale. Peanut-, Ara h 2-, and Ara h 6-specific IgE and peanut-specific IgG4 were analyzed using right-censored log-normal mixed-effects models with participant-specific random intercepts. No imputation was performed for missing biomarker measurements. Mixed-effects models used all available observations for each participant. Sample size at each assessment period is reported in the corresponding tables and figures. For each biomarker, the five follow-up-vs.-baseline contrasts were adjusted for multiplicity using the Holm procedure.

Cross-sectional exploratory associations were evaluated using Pearson's correlation analysis, as specified in individual figure legends. For analyses involving measurements that exceeded the assay upper limit, correlation results based on assay-limit values were considered descriptive.

Categorical variables were compared using Fisher's exact test. Receiver operating characteristic (ROC) analysis was used to assess discrimination of continuous candidate biomarkers. The identified thresholds were evaluated as exploratory candidate thresholds rather than validated diagnostic cutoffs in this study. Diagnostic-performance estimates were reported with confidence intervals where applicable.

An exploratory time-lagged analysis was performed to evaluate whether biomarker measurements at an earlier time point were associated with subsequent clinical desensitization. Candidate biomarker measurements were compared with the SCD achieved during a subsequent follow-up period.

All tests were two-tailed, and a *p* value < 0.05 was considered statistically significant, * *p* < 0.05, ** *p* < 0.01, *** *p* < 0.001. The mixed-effects, censored mixed-effects, Holm contrasts, and bootstrap CIs were performed in STATA 18.0, while GraphPad Prism 10 and SRplot (29) were used for BAT AUC calculations, Pearson correlation analysis, Fisher's exact test, ROC analyses.

## Results

### High desensitization rates and favorable safety

Of 49 patients who initiated clinical peanut OIT at IAA, 9 had incomplete OIT follow-up, including 5 patients who were lost to follow-up and counted as nonattainment, and were excluded from biomarker analytic cohort. Among 40 patients with adequate OIT follow-up, 8 had fewer than two BAT assessments performed ≥6 months apart and also excluded from the biomarker study (Figure 1A). Therefore, 32 patients aged 4–18 years met all screening criteria and were included in this longitudinal biomarker-analysis cohort study, with 16 having high baseline Ara h 2-sIgE (≥40 kU/L). The cohort was male-predominated in both high (75%) and low (81%) sIgE subgroups (Table 1). While mean total IgE level was higher in the high-sIgE subgroup, this difference was not statistically significant. The descriptive mean baseline Ara h 2-sIgE was 80.3 kU/L, with observed values ranging from 42.1 to ≥100 kU/L. The majority of patients with high Ara h 2-sIgE also had elevated peanut sIgE (15/16, Table 1). Ara h 2-sIgE and Ara h 6-sIgE showed strong and medium descriptive correlations with peanut sIgE, respectively (Supplementary Figure S2). Because several values exceeded the assay upper limit (100 kU/L), these correlations were interpreted descriptively. The true mean sIgE levels in high-sIgE group are likely underestimated.

**Table 1:** Demographic and clinical characteristics of two patient groups undergoing pOIT.

Characteristics	Ara h 2 sIgE ≥40	Ara h 2s IgE < 40	*p-value*
Demographics			
Total *n*	16	16	*ns*
Gender (M/F)	12/4	13/3	*ns*
Age (years, mean/range)	14.6 (10–18)	12.5 (4–18)	*ns*
Baseline Biomarkers (mean ± SEM)			
Total IgE (kU/L)	1,129.8 ± 305.4	471.9 ± 159.2	0.077*
Peanut sIgE (kU/L)	92.2 ± 4.0	30.5 ± 6.9	<0.001*
*n* (Peanut sIgE > 50 kU/L)	15	3	<0.001*
Ara h 2-sIgE (kU/L)	80.3 ± 5.4	15.0 ± 3.4	<0.001*
Ara h 6-sIgE (kU/L)	64.2 ± 7.8	12.7 ± 3.5	<0.001*
Peanut sIgG4 (mg/L)	0.7 ± 0.2	0.5 ± 0.1	*ns*
BAT AUC	254.1 ± 53.4	235.8 ± 60.9	*ns*
OIT Clinical Course			
OIT duration (months, mean ± SEM)	26.3 ± 1.7	30.1 ± 1.9	*ns*
*n* (OIT up dosing phase)	12	11	*ns*
*n* (OIT maintenance phase)	4	5	*ns*
SCD (mg, mean ± SEM, range)	771.1 ± 194.1 (36–3,000)	970.0 ± 423.3 (18–7,000)	ns
*n* (%) with SCD ≥1,000 mg	4 (25.0%)	5 (31.3%)	*ns*
*n* (%) with SCD ≥300 mg	11 (68.8%)	10 (62.5%)	*ns*
*n* (anaphylactic reaction during OIT)	1	1	*ns*

BAT, basophil activation test; AUC, area under the curve; SCD, successfully consumed dose daily; OIT, peanut oral immunotherapy.

*statistically significant difference.

Compared with the commercial Palforzia® protocol, a conservative, slow-escalation regimen was used (Supplementary Table S2), starting at an extremely low dose (0.1 mg peanut protein) and progressing through a prolonged up-dosing phase (≥18 clinic visits) to reach a target of ≥300 mg peanut protein (Figure 1A). Most scheduled doses were completed during the initial 18 visits, although a few missing doses occurred (Figure 1B), followed by individualized dosing (continuing escalation or maintenance) for up to 36 months (maximum 35 visits). The highest successfully consumed dose (SCD), assessed at about 6-month intervals, increased steadily through 24 months and remained stable for 25–36 months (Figure 1C). Treatment duration did not differ significantly between subgroups; however, the study was not powered to establish differences in escalation time. The planned interval between dose-escalation visits was 14–28 days during the first 18 visits; however, the observed intervals were generally longer than planned (Figure 1D) and longer than the 14-day interval recommended for Palforzia® (6), reflecting a very-low-dose initiation with extended-interval, prolonged up-dosing protocol. All 32 participants completed the first 18 visits within 12–24 months. The speed of desensitization varied; the fastest patient reached 300 mg in 14 visits. By 24 months, effective desensitization –-defined as a SCD ≥300 mg— was achieved in 68.8% (11/16) of the high sIgE subgroup and 62.5% (10/16) of the lower sIgE subgroup (relative risk 1.10, 95% CI 0.67–1.82) (Table 1). Although observed desensitization proportions were numerically similar between the high- and lower-Ara h 2-sIgE subgroups, due to the wide confidence intervals, the study was not powered to establish therapeutic equivalence across sensitization strata. Most successfully desensitized patients continued dose escalation to further raise their tolerated daily dose; nine exceeded 1,000 mg, with the highest reaching 7,000 mg (Table 1). Notably, six of eight patients who completed OIT but were not included in this biomarker study attained SCD ≥300 mg. Therefore, among all 49 patients who initiated OIT, 27 (55.1%) had documented attainment of SCD ≥300 mg. Among all 40 evaluable patients with adequate OIT follow-up to ascertain dose progression, this rate was 67.5% (27 participants), which aligns with prior reports for conventional OIT (62.0%–67.2%) (7, 30) and low-dose OIT (74.2%) (14).

During pOIT, some patients experienced mild-to-moderate dosing-related adverse events, including mouth itch, transient abdominal pain, nausea and taste aversion (not systematically analyzed). Anaphylaxis occurred in one patient per subgroup (Table 1). No other protocol-defined severe adverse events, hospitalizations, or treatment discontinuations due to adverse reactions were reported and no cases of eosinophilic esophagitis were diagnosed.

Overall, no statistically significant differences between subgroups were detected in the observed desensitization or severe adverse-event outcomes under this protocol; however, the estimates were imprecise because of the small sample size. Because the subsequent biomarker analyses were designed to characterize longitudinal associations across the treated cohort, and no significant time-by-sensitization-group interaction was detected for SCD trajectories, the subgroups were pooled for exploratory biomarker analyses.

### Effective peanut OIT leads to longitudinal suppression of basophil activity

Given the heterogeneous dose-response patterns characteristic of BAT (31) (Supplementary Figure S3), the area under the curve (AUC) was used as the primary metric for basophil activity, as it showed close descriptive concordance with maximum dose response (MDR) (Supplementary Figure S4). To assess whether higher peanut sIgE levels are associated with increased basophil reactivity, baseline peanut sIgE levels were compared with available baseline BAT AUC in 25 patients (Baseline BAT was unavailable in 7 participants because their first BAT measurement occurred after OIT initiation). No significant correlation was observed (*p* = 0.42) (Supplementary Figure S5A), and baseline basophil activity did not statistically differ between two groups (Table 1). Moreover, baseline BAT AUC was not associated with desensitization outcome (*p* = 0.76) (Supplementary Figure S5B), and no predictive cutoff could be identified (Supplementary Figure S5C).

To evaluate basophil activation dynamics during pOIT, each patient underwent at least two BAT measurements spaced ≥6 months apart. In contrast to some prior reports (23), our protocol induced a transient increase in basophil activity—measured by peanut-induced %CD63 basophils (AUC and MDR%)—during months 2–6 of the build-up phase, followed by a progressive and significant decline (Table 2, Figure 2A). In mixed-effects analysis accounting for within-participant repeated measurements and multiplicity, BAT activity showed a significant overall change over time (Table 3, *p* = 7.65 × 10⁻^7^), with statistically significant suppression relative to baseline emerging at 19–24 months (Holm-adjusted *p* = 0.037) and becoming more pronounced at 25–36 months (Holm-adjusted *p* = 0.00028). A similar trajectory was observed in the high Ara h 2-sIgE subgroup (Figure 2B, Table 3). It has to be mentioned that one patient had a pre-OIT BAT with an anti-IgE-negative/fMLP-positive pattern consistent with a non-releaser phenotype. This measurement was excluded from all BAT-specific analyses and was not imputed. Subsequent BAT assessments from the same participant obtained during OIT demonstrated anti-IgE-positive/fMLP-positive responses and were therefore included in the analyses.

**Table 2:** Descriptive longitudinal changes of biomarkers in response to peanut OIT.

Biomarker	0 M	2–6 M	7–12 M	13–18 M	19–24 M	25–36 M
Maximum SCD^a^ (mg, mean ± SEM)						
Peanut protein	0.1 ± 0.01	5.7 ± 2.3	72.5 ± 21.6	439.8 ± 218.6	705.1 ± 250.7	721.3 ± 162.1
BAT (mean ± SEM)						
AUC (Peanut)	243.8 ± 45.3	357.4 ± 62.7	304.6 ± 54.6	207.0 ± 31.6	123.7 ± 28.4	58.8 ± 22.9
*n* (%) (AUC <100)	11/25 (44%)	2/15 (13%)	3/17 (18%)	5/19 (26%)	12/21 (57%)	10/11 (91%)
MDR (%CD63 + )	36.6 ± 5.2	47.5 ± 6.1	33.0 ± 6.1	27.3 ± 4.6	18.4 ± 4.3	10.4 ± 3.7
IgE (kU/L, mean ± SEM)^b^						
Total IgE (tIgE)	790.3 ± 180.5	1,178.6 ± 256.0	1,081.4 ± 229.5	894.4 ± 181.3	1,058.8 ± 204.3	1,581.5 ± 489.5
Peanut sIgE	61.3 ± 6.8	97.7 ± 1.5	67.8 ± 8.4	64.0 ± 8.7	57.0 ± 8.2	31.0 ± 8.2
*n* (%) (Peanut sIgE > 50)	18/32 (56%)	18/18 (100%)	14/21 (67%)	13/21 (62%)	10/20 (50%)	3/15 (20%)
Ara h 2sIgE	47.7 ± 6.7	85.2 ± 4.3	55.6 ± 8.7	49.9 ± 8.9	42.6 ± 8.3	20.4 ± 7.1
*n* (%) (Ara h 2-sIgE > 40)	16/32 (50%)	18/18 (100%)	13/21 (62%)	13/20 (65%)	9/20 (45%)	2/15 (13%)
Ara h 6sIgE	38.4 ± 6.2	76.4 ± 7.0	47.8 ± 8.0	41.8 ± 8.6	34.7 ± 7.0	16.6 ± 5.4
sIgE/tIgE Ratio (mean ± SEM)						
Peanut	14.1% ± 2.0%	15.7% ± 2.9%	11.3% ± 2.4%	13.1% ± 2.9%	10.3% ± 2.9%	8.1% ± 3.0%
Ara h 2	10.0% ± 1.7%	13.3% ± 2.5%	9.8% ± 2.3%	9.5% ± 2.3%	8.0% ± 2.9%	6.3% ± 2.7%
Ara h 6	7.7% ± 1.4%	9.1% ± 1.7%	8.0% ± 1.9%	7.6% ± 2.1%	5.6% ± 2.0%	5.1% ± 2.2%
sIgG4 (mg/L, mean ± SEM)^b^						
Peanut	0.6 ± 0.1	1.5 ± 0.4	2.8 ± 0.8	4.5 ± 1.2	8.1 ± 2.7	7.8 ± 2.4
Ara h 2	0.3 ± 0.0	0.6 ± 0.3	1.0 ± 0.3	2.2 ± 0.8	6.9 ± 2.8	5.7 ± 2.4
Ara h 6	0.3 ± 0.0	0.8 ± 0.2	1.7 ± 0.6	2.7 ± 1.1	7.9 ± 2.8	7.7 ± 3.1
sIgE/sIgG4 Ratio^c^ (mean ± SEM)						
Peanut	149.1 ± 22.4	141.7 ± 34.9	80.6 ± 35.1	52.7 ± 13.6	46.1 ± 27.3	6.5 ± 2.9
*n* (%) (Ratio < 50)	6/27 (22%)	3/11 (27%)	6/12 (50%)	13/18 (72%)	10/12 (83%)	6/6 (100%)
Ara h 2	184.4 ± 25.4	261.0 ± 26.2	148.3 ± 44.4	104.4 ± 22.9	74.4 ± 38.5	11.0 ± 4.5
Ara h 6	182.4 ± 24.3	196.4 ± 32.0	114.8 ± 49.4	99.7 ± 22.6	48.4 ± 32.2	6.3 ± 3.0

a SCD: successfully consumed total daily peanut-protein dose; values shown are the maximum SCD recorded within each assessment period.

b For descriptive summaries in Table 2 and graphical presentation, sIgE and sIgG4 values exceeding the upper limits of quantification (100 kU/L and 30 mg/L, respectively) were displayed at the assay limits. For inferential longitudinal analyses, these observations were treated as right-censored.

c The values from the lab test reports were directly used to calculate the sIgE/sIgG4 [(kU/L)/(mg/L)] ratio.

**Figure 2:**
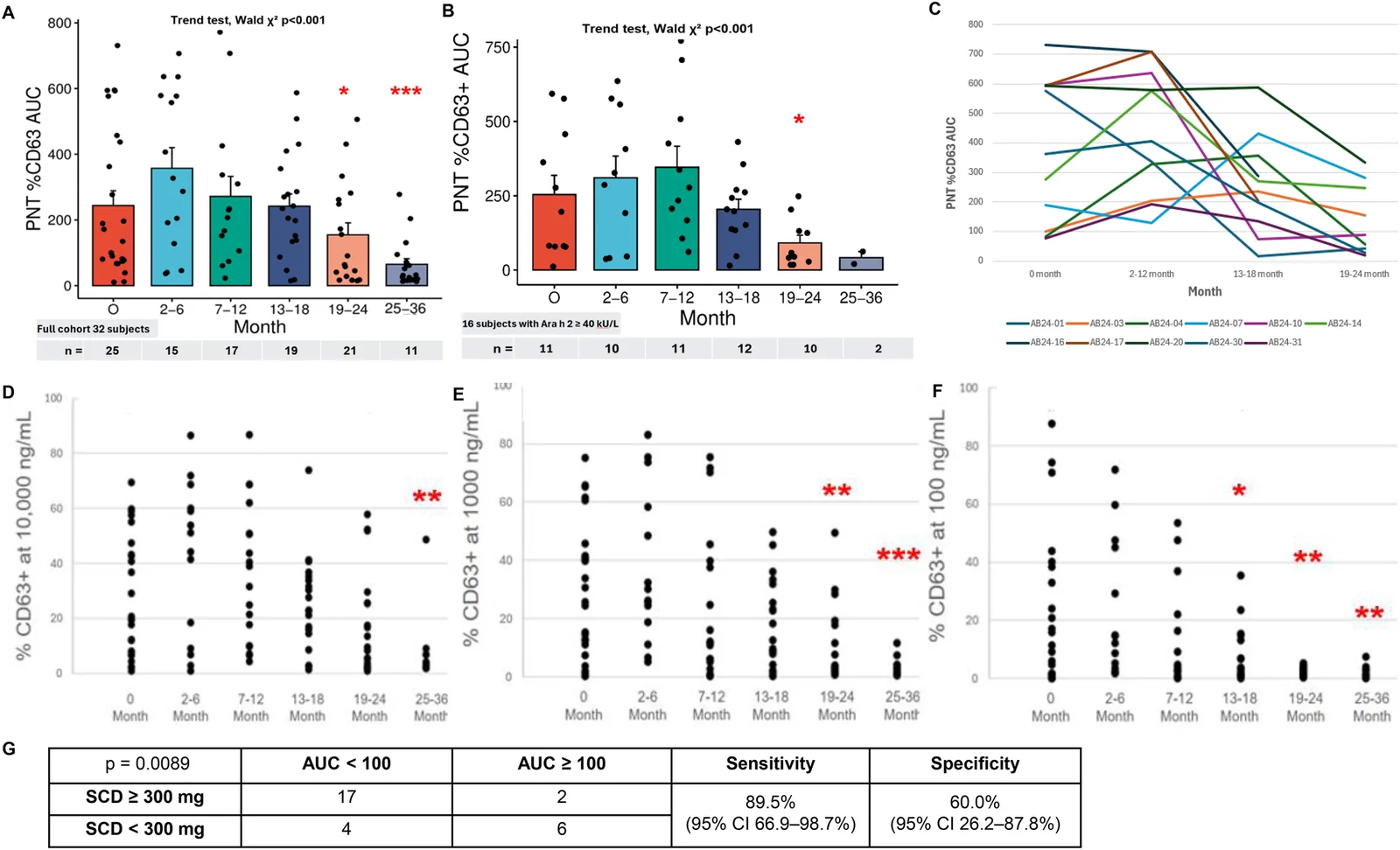
Progressive inhibition of basophil activation during peanut oral immunotherapy. **(A)** Basophil activation, quantified as the basophil activation test (BAT) area under the curve (AUC), showed a biphasic pattern in the full cohort (*n* = 32), with transient early enhancement during months 2–6 followed by progressive suppression during extended up-dosing and maintenance. The overall time effect was significant (Wald *χ*^2^, *p* < 0.001). **(B)** Patients with high baseline Ara h 2-specific IgE (sIgE ≥40 kU/L; *n* = 16) showed a similar longitudinal pattern. **(C)** Representative **i**ndividual longitudinal trajectories of basophil activation show variable baseline activity and heterogeneous responses to peanut OIT over time. **(D–F)** BAT responses to peanut stimulation at 10,000, 1,000, and 100 ng/mL, respectively, showed progressive reductions in the percentage of CD63⁺ basophils during treatment. Significant suppression was first observed at 13–18 months at 100 ng/mL, followed by 19–24 months at 1,000 ng/mL and 25–36 months at 10,000 ng/mL. **(G)** In an exploratory patient-level analysis (*n* = 29), the last available BAT AUC during months 13–36 was compared with the maximum SCD achieved during the same period. BAT AUC <100 was associated with SCD ≥300 mg (Fisher's exact *p* = 0.0089), with sensitivity 89.5% (95% CI, 66.9–98.7%) and specificity 60.0% (95% CI, 26.2–87.8%). Data are presented as mean ± SEM for panels **(A,B)**. Global time-effect *p* values from longitudinal mixed-effects model analyses (trend tests) are shown above panel **(A,B)**. *p < 0.05, **p < 0.01, ***p < 0.001 for Holm-adjusted pairwise comparisons with baseline.

**Table 3:** Estimated marginal means from longitudinal mixed-effects models by assessment period.

Biomarker	Baseline	2–6 months	7–12 months	13–18 months	19–24 months	25–36 months
BAT AUC	140.35* (82.92–197.78)**	219.77 (106.19–333.36)	202.89 (104.00–301.77)	140.05 (75.17–204.93)	68.53 (38.20–98.86)	35.54 (14.32–56.76)
PNT sIgE	36.74 (18.02–55.47)	49.46 (23.34–75.57)	41.79 (19.97–63.61)	41.64 (19.90–63.38)	32.61 (15.53–49.70)	19.88 (9.08–30.68)
Ara h 2-sIgE	21.01 (7.94–34.08)	34.84 (12.02–57.67)	25.26 (8.98–41.54)	26.55 (9.35–43.75)	18.94 (6.67–31.21)	8.88 (2.94–14.83)
Ara h 6-sIgE	15.86 (5.25–26.48)	28.75 (8.69–48.82)	20.23 (6.39–34.06)	17.48 (5.44–29.53)	14.52 (4.48–24.56)	6.46 (1.90–11.01)
PNT sIgG4	0.51 (0.33–0.69)	1.00 (0.51–1.50)	1.60 (0.84–2.36)	2.32 (1.47–3.17)	3.93 (2.01–5.86)	5.08 (2.59–7.56)
PNT sIgE/sIgG4 ratio	67.50 (27.03–107.97)	57.74 (14.80–100.68)	24.65 (6.69–42.61)	23.36 (8.06–38.66)	8.75 (2.39–15.10)	4.21 (0.97–7.45)

Estimates were obtained from mixed-effects models with assessment period as a categorical fixed effect and participant-specific random intercepts. Values are back-transformed estimates with 95% confidence intervals. They are not expected to equal the arithmetic means reported descriptively in Table 2. sIgE and sIgG4 were analyzed using right-censored log-normal mixed-effects models. The sIgE/sIgG4 ratio was calculated from reported laboratory values and was analyzed as an exploratory secondary outcome. Global time effects were significant (*p* < 0.001) for all listed outcomes; follow-up-vs.-baseline contrasts were adjusted using the Holm procedure. The unit for sIgE is kU/L and for sIgG4 is mg/L.

* Means.

** 95% confidence intervals.

BAT serial peanut stimulation revealed a stepwise reduction in CD63⁺ basophils over time. Significant suppression was first observed at 13–18 months at low peanut concentration (100 ng/mL), followed by intermediate (1,000 ng/mL) at 19–24 months, and high concentration (10,000 ng/mL) at 25–36 months (Figures 2D–F). These findings indicate a progressive increase in the basophil activation threshold during pOIT, aligned with the rise in SCD (Figure 1C). Notably, even at 25–36 months—when effective desensitization is achieved—basophils remain responsive but require higher allergen doses for activation.

Despite an overall downward trend following the initial increase, substantial inter-patient variability was observed in both baseline BAT levels and longitudinal response patterns (Figure 2C). BAT AUC at 19–24 months did not linearly correlate with treatment effectiveness. Among participants with evaluable BAT measurements, although 11/25 (44.0%) had a baseline BAT AUC <100 before OIT, BAT AUC ≥100 was observed during the up-dosing phases in 13/15 (86.7%) patients at 2–6 months and 14/17 (82.4%) patients at 7–12 months (Figure 2A, Table 2). When the last available BAT AUC during months 13–36 was compared with the participant's maximum SCD achieved during the same period, a BAT AUC <100 was significantly associated with effective desensitization (SCD ≥300 mg) (Figure 2G) in an exploratory patient-level analysis, with a sensitivity of 89.5% (95% CI, 66.9–98.7%) and specificity of 60.0% (95% CI, 26.2–87.8%). These findings suggest that longitudinal BAT suppression may reflect acquisition of clinical desensitization; however, the same-cohort threshold is an exploratory study-derived threshold. Its performance was not internally validated and is likely optimistic. The low specificity (only 60%) indicated that an AUC <100 alone is insufficient to distinguish patients who achieved effective desensitization. Consistent with this limitation, continuous ROC analysis yielded an AUC of 0.784 (95% CI, 0.536–0.983), with the wide confidence interval reflecting limited precision. The observed cutoff should be considered a hypothesis-generating candidate threshold and requires validation in larger independent prospective studies.

### Peanut OIT slowly reduces peanut and Ara h 2/6-sIgE levels

Longitudinal changes of sIgE against peanut and its major components (Ara h 2 and Ara h 6) were evaluated. Consistent with prior reports (23), peanut sIgE levels demonstrated a biphasic pattern, with an early increase during months 2–6 (100% of patients exceeding 50 kU/L for peanut sIgE), followed by a gradual decline at later time points (Table 2, Figures 3A–D). However, the sIgE/total IgE ratio remained generally stable over time, with no significant overall longitudinal time effect (Figure 3B). Mixed-effects analysis confirmed a significant overall effect of time for peanut sIgE, with an early increase followed by a later decline (Table 3). Notably, the rate of decline was slower than that reported in other pOIT protocols (15), and significant reductions from baseline were observed only after 25–36 months (Holm-adjusted *p* = 0.0063). This delay may be partly attributable to censoring of values >100 kU/L in real-world laboratory measurements. Notably, at 19–24 months, a substantial proportion of patients retained elevated Ara h 2-sIgE despite achieving clinical desensitization (Figure 3C bottom), indicating that serologic IgE decline lagged behind functional and clinical improvement. Peanut sIgE levels at 19–36 months did not correlate with SCD (data not shown).

**Figure 3:**
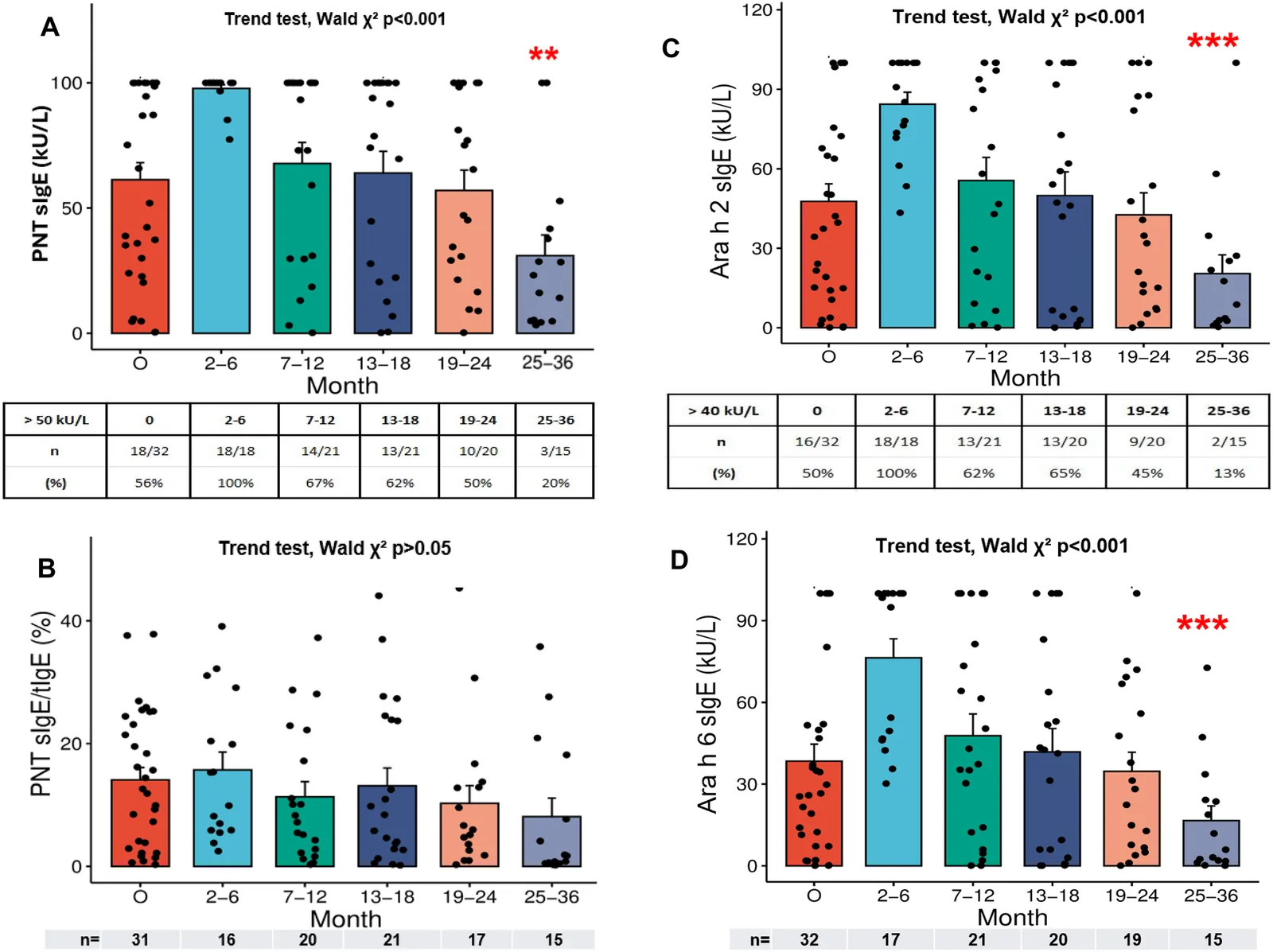
Gradual and delayed changes in peanut-specific IgE levels and the sIgE/tIgE ratio during peanut oral immunotherapy. For graphical presentation, values exceeding the upper limit were displayed at the assay upper limit; for inferential longitudinal analyses, these values were treated as right-censored. **(A)** Peanut-specific IgE (sIgE) initially increased, followed by a gradual downward trend over the course of OIT; a statistically significant reduction was observed only at 25–36 months. **(B)** The peanut sIgE/total IgE (tIgE) ratio remained largely stable throughout OIT. **(C,D)** Component-specific IgE to Ara h 2 and Ara h 6 exhibited longitudinal patterns similar to those observed for peanut sIgE. Data are presented as mean ± SEM. Right-censored observations may underestimate baseline levels and attenuate observed changes in highly sensitized individuals. Global time-effect *p* values from longitudinal mixed-effects model analyses (trend tests) are shown above the corresponding panels. * *p* < 0.05, **p < 0.01, *** *p* < 0.001 for Holm-adjusted pairwise comparisons with baseline.

### Peanut- and Ara h 2/6-specific sIgG4 increase early and significantly during pOIT

In contrast to sIgE, sIgG4 levels against peanut and its components increased early and significantly (Table 2 and Figure 4A). The right-censored log-normal mixed-effects model showed a significant increase already at 2–6 months, ratio ≈ 1.96, adjusted *p* = 0.0095, with progressively larger increases thereafter (Table 3). Notably, at the cohort level, mean peanut sIgG4 increased over time while mean BAT AUC declined, demonstrating opposing longitudinal trajectories (Figure 5). Peanut-specific sIgG4 at 13–24 months did not differ significantly according to baseline BAT category (Supplementary Figure S6).

**Figure 4:**
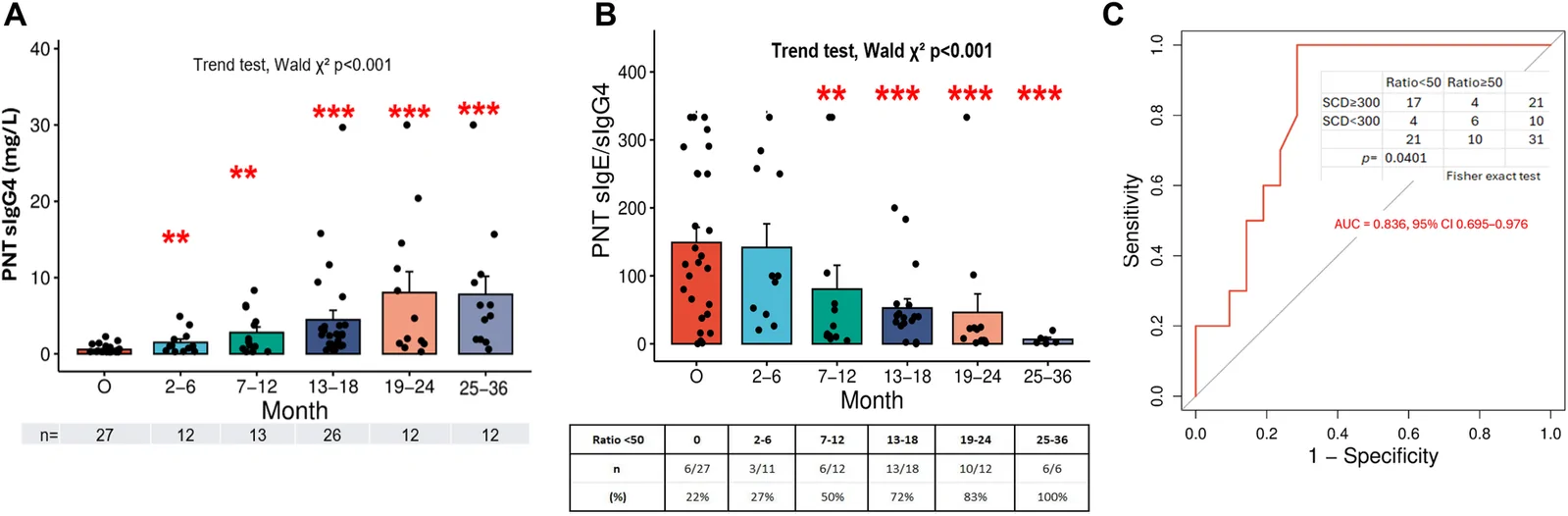
Early increase in peanut-specific IgG4 and reduction in the sIgE/sIgG4 ratio during peanut oral immunotherapy. **(A)** Values exceeding the sIgG4 upper limit were displayed at the assay limit for graphical presentation and treated as right-censored in inferential longitudinal analysis. Peanut-specific IgG4 (sIgG4) increased as early as 2–6 months, continued rising through up-dosing and maintenance phases. Component sIgG4 showed similar trends (data not shown). Right-censored observations may underestimate the magnitude of responses in highly responsive individuals. **(B)** The peanut sIgE/sIgG4 index was calculated directly from laboratory-reported values and analyzed as an exploratory secondary outcome. The sIgE/sIgG4 ratio progressively decreased during pOIT, with minimum values <50 observed in 13 of 18 participants (72%) at 13–18 months. **(C)** The lowest post-treatment peanut sIgE/sIgG4 ratio <50 was associated with SCD ≥300 mg in an exploratory dichotomous outcome-stratification analysis (*n* = 31). Data are presented as mean ± SEM for panels **(A,B)**. * *p* < 0.05, **p < 0.01, *** *p* < 0.001 for Holm-adjusted pairwise comparisons with baseline derived from the longitudinal mixed-effects model.

**Figure 5:**
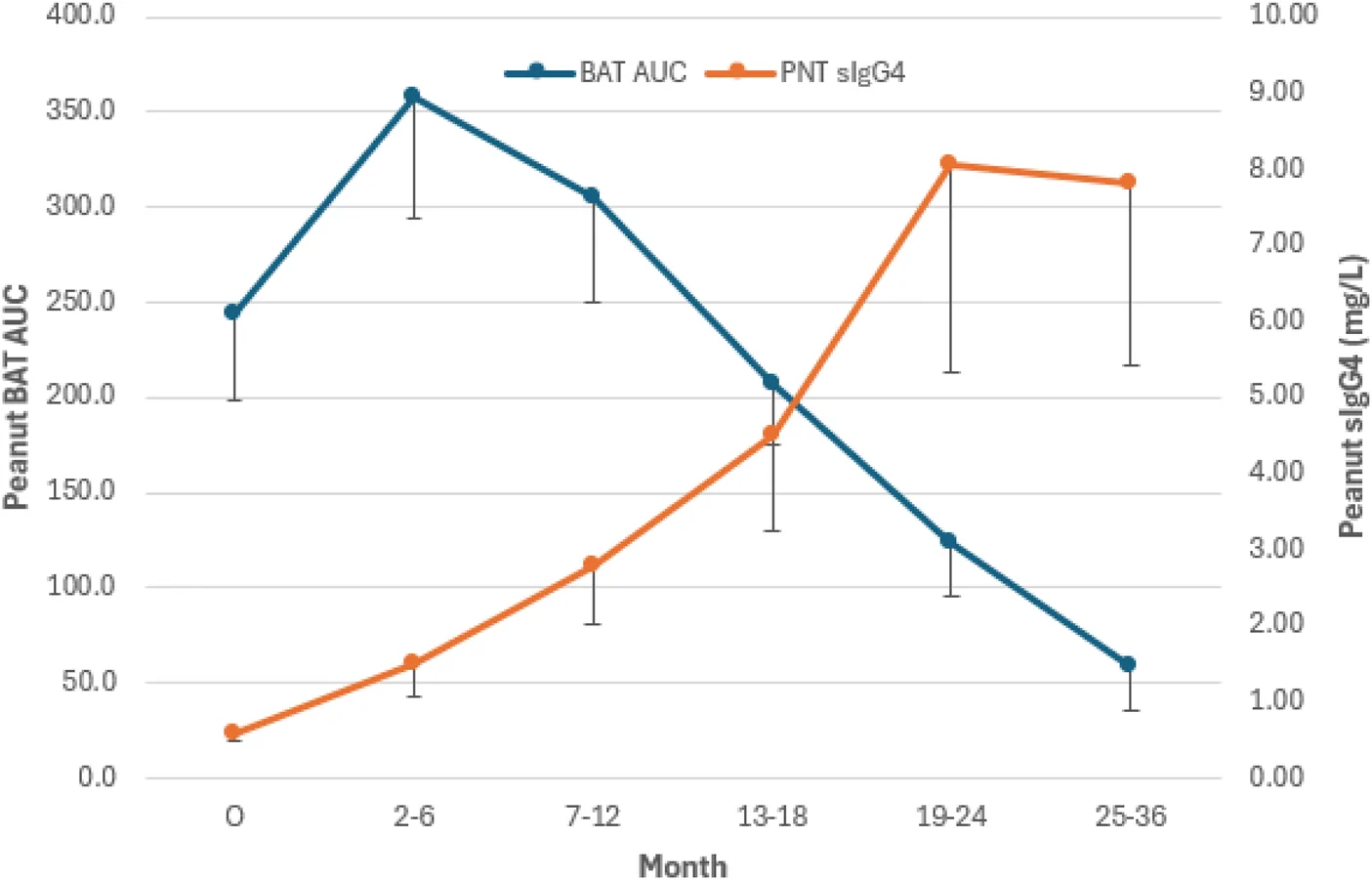
Inverse temporal trajectories of peanut-specific IgG4 and basophil activation during peanut oral immunotherapy. Mean peanut sIgG4 increased over time while mean BAT AUC decreased, demonstrating opposing longitudinal trajectories. Data are presented as mean ± SEM. This cohort-level comparison is descriptive and is not intended to establish an independent patient-level association.

Allergen-specific IgG4 functions as a blocking antibody by competing with IgE for allergen binding during OIT. Accordingly, the sIgE/sIgG4 ratio may provide a more integrated marker of treatment response. As shown in Figure 4B, this ratio decreased progressively during therapy, with minimum values <50 observed in 13 of 18 participants (72%) at 13–18 months. Although the lowest post-treatment peanut sIgE/sIgG4 ratio did not show a linear correlation with desensitization thresholds (*n* = 31, *R*^2^ = 0.1066, *p* = 0.07), it demonstrated apparent discriminatory performance in ROC analysis (AUC = 0.836, 95% CI 0.695–0.976). An exploratory dichotomous cutoff of 50 yielded a positive predictive value (PPV) of 80.9% and a negative predictive value (NPV) of 60.0% for effective desensitization (SCD ≥300 mg) (Figure 4C). Given the small cohort, its diagnostic performance is likely optimistic. The ratio <50 threshold was exploratory and was evaluated in the same cohort in which it was identified; therefore, it requires independent validation. For clinical applicability, raw laboratory values (kU/L for sIgE, mg/L for sIgG4) were used directly to calculate this ratio without unit conversion (Table 2).

Finally, we performed an exploratory time-lagged analysis to assess whether biomarker measurements at 13–18 months were associated with subsequent successful clinical desensitization (SCD ≥300 mg) at 19–24 months. These intervals were selected based on the longitudinal trajectories observed in our cohort: BAT suppression first became evident during 13–18 months, the sIgE/sIgG4 ratio had substantially declined by this period, and SCD increased substantially during 19–24 months, crossing the 300-mg clinical threshold at the cohort level. For BAT AUC (*n* = 17), the apparent AUC was 0.788, but the estimate was imprecise, with a wide bootstrap 95% CI 0.54–1.00. The BAT AUC <100 threshold did not significantly discriminate subsequent SCD ≥300 mg (Fisher's exact test, *p* = 0.102). For the sIgE/sIgG4 ratio (*n* = 18), the lagged AUC was 0.569 with a bootstrap 95% CI 0.28–0.84, and a ratio <50 did not significantly discriminate subsequent SCD ≥300 mg. Overall, these exploratory time-lagged analyses did not provide sufficiently precise evidence to establish prospective predictive utility.

## Discussion

In this retrospective real-world cohort, clinically meaningful increases in ingested peanut dose were observed during a low-dose initiation, slow-escalation pOIT protocol in children with high Ara h 2-sIgE, a population traditionally considered high risk. Observed desensitization proportions and severe adverse-event frequencies did not differ significantly between sensitization subgroups, although the study was not designed or powered to establish equivalence or comparative safety. The percentage of patients who had adequate OIT follow-up and achieved SCD ≥300 mg is consistent with published OIT outcomes. Baseline BAT and sIgE were not associated with clinical responses. In contrast, progressive reductions in basophil activation and shifts in the sIgE/sIgG4 balance consistently paralleled successful dose escalation during treatment. Specifically, longitudinal decreases in BAT AUC (<100) and in the sIgE/sIgG4 ratio (<50) were significantly associated with effective desensitization (SCD ≥300 mg) in exploratory analyses. These findings suggest that longitudinal immune trajectories may provide more informative measures of treatment-associated immune changes than baseline biomarker measurements.

High baseline sIgE has historically been linked to increased adverse reactions, slower desensitization, and higher dropout rates, leading to its use as an exclusion criterion in earlier guidelines (e.g., EAACI 2017) (32). The 40 kU/L Ara h 2-sIgE threshold was selected to characterize marked sensitization based on published clinical data (25) and should not be considered a universal OIT-response threshold. Because Ara h 2 distributions and diagnostic performance may vary across populations and geographic settings, the clinical applicability of this threshold may differ. Our findings therefore primarily support the feasibility of clinically meaningful desensitization under this conservative protocol in patients with high Ara h 2-sIgE, rather than equivalence between sensitization strata. The study also cannot determine whether this protocol reduced risk relative to other OIT approaches. The low-dose initiation, slow escalation, and individualized maintenance approach produced outcomes consistent with prior reports using conventional OIT approaches (7, 9). These findings support the practical value of prioritizing tolerability and gradual dose escalation, although whether such an approach improves long-term adherence or treatment persistence requires prospective evaluation. Real-world adherence and treatment persistence are important determinants of the effectiveness of peanut OIT. Recent real-world experience with food-based OIT has similarly highlighted treatment discontinuation and loss to follow-up as important challenges (33). In our cohort, the observed high desensitization rate should be interpreted in the context of the biomarker-analytic cohort, which required completion of OIT and at least two BAT assessments and may therefore have preferentially excluded patients who discontinued treatment early. Although missed doses and dosing-related reactions were documented during treatment, long-term adherence after the observation period was not systematically assessed.

Peanut OIT induces characteristic immunologic shifts. Allergen-specific IgE typically rises early and then declines gradually during maintenance, but elevated IgE may persist in some patients (19, 23). In our cohort, IgE decline lagged behind clinical and functional improvement, with 45% of participants retaining Ara h 2-sIgE ≥40 kU/L at 19–24 months despite achieving desensitization. In contrast, allergen-specific IgG4 increased early, with increase in peanut and Ara h 6-sIgE evident as early as 2–6 months. Given the ability of IgG4 to competitively inhibit IgE-mediated activation (34), the sIgE/sIgG4 ratio provides an integrated measure of the balance between allergen-specific IgE and IgG4. We observed a progressive decline in this ratio during OIT, and an exploratory ratio <50 was associated with effective desensitization, supporting its potential utility as a practical adjunctive measure of treatment-associated immune change.

Basophil activation provides a functional measure of allergen-induced effector-cell responsiveness. Although prior studies have reported associations between baseline BAT responsiveness and OIT outcomes (22, 23), and that participants with low baseline basophil responsiveness are more likely to achieve treatment success, baseline BAT was not associated with desensitization in our cohort. Longitudinal BAT assessment instead revealed a biphasic pattern, with transient activation during early up-dosing (months 2–6) followed by progressive suppression during extended dosing and maintenance. This trajectory paralleled the gradual increase in tolerated dose and the longitudinal changes observed in humoral biomarkers. Importantly, subsequent progressive reduction in basophil activation, including BAT AUC <100, accompanied by an increased activation threshold, was associated with effective desensitization in an exploratory patient-level analysis. However, the <100 threshold was derived and evaluated in the same cohort and should therefore be considered hypothesis-generating and requires validation in an independent cohort. Longitudinal BAT may thus provide a complementary functional measure of treatment-associated immune modulation, although its clinical utility remains to be established prospectively.

Collectively, these findings highlight fundamental limitations of relying on static baseline biomarkers alone. Rather than demonstrating prospective predictive value, our results indicate that longitudinal changes in BAT and the sIgE/sIgG4 ratio were associated with increasing tolerated dose during pOIT. These dynamic measures may therefore provide adjunctive information about treatment-associated immune changes and complement clinical assessment during dose escalation and maintenance.

These observations have potential clinical implications. If prospectively validated against standardized oral food challenges and in independent cohorts, longitudinal BAT and sIgE/sIgG4 trajectories may provide adjunctive information during clinical assessment of pOIT. Early increases in sIgE and BAT activity were observed in some participants despite subsequent increases in tolerated dose, indicating that early biomarker changes should not be interpreted in isolation. Continued longitudinal assessment may provide a more informative context for interpreting these dynamic immune changes during treatment and for counseling patients and families.

### Limitations

This study has several limitations. Its single-center, retrospective design and modest sample size may limit generalizability and statistical power for subgroup analyses. It lacked an untreated, placebo, or alternative OIT-protocol comparator; therefore, the contribution of the low-dose, slow-escalation strategy itself cannot be isolated from the effects of OIT more generally. Eligibility required at least two BAT assessments separated by ≥6 months, which may have preferentially excluded patients who discontinued treatment early and could introduce selection bias. Biomarker availability varied across assessment periods. No imputation was performed, and mixed-effects analyses used all available observations. Clinical desensitization was defined using the successfully consumed dose during routine care rather than a standardized blinded oral food challenge; therefore, the candidate biomarkers were not validated against the diagnostic gold standard.

The BAT assay was developed by study authors, and although laboratory and clinical data generation incorporated internal blinding and separation of responsibilities, no independent external reanalysis was performed. AUC measurement uncertainty remains unquantified. Dichotomous cutoff values (AUC <100, sIgE/sIgG4 ratio <50) are exploratory and require validation in large independent cohorts. In addition, patient-reported outcomes, including quality-of-life measures, were not systematically collected and represent an important area for future studies. Long-term adherence was not systematically assessed, and sustained unresponsiveness after treatment discontinuation was not evaluated. Finally, the exclusion of children under 4 years leaves unanswered questions regarding biomarker utility in the population most likely to benefit from disease-modifying intervention. Prospective studies are needed to determine whether longitudinal biomarker assessment provides clinically useful information and whether this OIT approach improves safety, adherence, or treatment outcomes relative to alternative protocols.

The calendar-time assay-control analysis also has limitations. Available longitudinal control data covered January 2024 through July 2026 and therefore did not include the earlier study period from September 2021 through December 2023. In addition, the linear trend analysis was designed to detect an overall monotonic temporal trend and may not capture nonlinear variation in control responses. Finally, anti-IgE and fMLP positive controls assess basophil responsiveness and general assay performance but do not directly detect lot-specific changes in the peanut stimulation reagent.

## Conclusions

In this retrospective cohort, clinically meaningful increases in ingested peanut dose were observed in participants with both high and lower baseline Ara h 2-sIgE, although equivalence between sensitization subgroups was not established. Longitudinal suppression of basophil activation and reductions in the sIgE/sIgG4 ratio were associated with higher SCD, whereas baseline biomarkers and exploratory time-lagged analyses did not establish prospective predictive utility. These findings support further prospective evaluation of BAT and antibody trajectories as adjunctive biomarkers during pOIT, with validation against standardized oral food challenges and in independent cohorts required before clinical implementation.

## Data Availability

The raw data supporting the conclusions of this article will be made available by the authors, without undue reservation.

## References

[1] Sindher SB Nadeau KC Chinthrajah RS Leflein JG Bégin P Ohayon JA. Efficacy and safety of dupilumab in children with peanut allergy: a multicenter, open-label, phase II study. Allergy. (2025) 80(1):227–37. 10.1111/all.16404 39673452 PMC11724241

[2] Gupta RS Lau CH Hamilton RG Donnell A Newhall KK. Predicting outcomes of oral food challenges by using the allergen-specific IgE-total IgE ratio. J Allergy Clin Immunol Pract. (2014) 2(3):300–5. 10.1016/j.jaip.2013.12.006 24811021

[3] ASCIA. Position Paper—Oral Immunotherapy for Food Allergy. Australasian Society of Clinical Immunology and Allergy (2024); Available online at: https://www.allergy.org.au/hp/papers/ascia-oral-immunotherapy-for-food-allergy (Accessed December 30, 2025).

[4] Pouessel G Lezmi G. Oral immunotherapy for food allergy: translation from studies to clinical practice? World Allergy Organ J. (2023) 16(2):100747. 10.1016/j.waojou.2023.100747 36816599 PMC9932561

[5] Santos AF Riggioni C Agache I Akdis CA Akdis M Alvarez-Perea A. EAACI guidelines on the management of IgE-mediated food allergy. Allergy. (2025) 80(1):14–36. 10.1111/all.16345 39473345 PMC11724237

[6] Investigators PGoC, Vickery BP Vereda A Casale TB Beyer K Du Toit G. AR101 oral immunotherapy for peanut allergy. N Engl J Med. (2018) 379(21):1991–2001. 10.1056/NEJMoa1812856 30449234

[7] de Silva D Rodríguez del Río P De Jong NW Khaleva E Singh C Nowak-Wegrzyn A. Allergen immunotherapy and/or biologicals for IgE-mediated food allergy: a systematic review and meta-analysis. Allergy. (2022) 77(6):1852–62. 10.1111/all.15211 35001400 PMC9303769

[8] Jones SM Kim EH Nadeau KC Nowak-Wegrzyn A Wood RA Sampson HA. Efficacy and safety of oral immunotherapy in children aged 1–3 years with peanut allergy (the immune tolerance network IMPACT trial): a randomised placebo-controlled study. Lancet. (2022) 399(10322):359–71. 10.1016/S0140-6736(21)02390-4 35065784 PMC9119642

[9] McHenry M Begin P Chan ES Latrous M Kim H. Food oral immunotherapy. Allergy Asthma Clin Immunol. (2024) 20(Suppl 3):82. 10.1186/s13223-025-00948-5 PMC11823072 39940042

[10] Chinthrajah RS Purington N Andorf S Long A O'Laughlin KL Lyu SC. Sustained outcomes in oral immunotherapy for peanut allergy (POISED study): a large, randomised, double-blind, placebo-controlled, phase 2 study. Lancet. (2019) 394(10207):1437–49. 10.1016/S0140-6736(19)31793-3 31522849 PMC6903389

[11] Loke P Wang X Lloyd M Ashley SE Lozinsky AC Gold M. Two-year post-treatment outcomes following peanut oral immunotherapy in the probiotic and peanut oral immunotherapy-003 long-term (PPOIT-003LT) study. Allergy. (2024) 79(10):2759–74. 10.1111/all.16262 39099231

[12] Baumert JL Taylor SL Koppelman SJ. Quantitative assessment of the safety benefits associated with increasing clinical peanut thresholds through immunotherapy. J Allergy Clin Immunol Pract. (2018) 6(2):457–65.e4. 10.1016/j.jaip.2017.05.006 28669889

[13] Blackman AC Staggers KA Kronisch L Davis CM Anagnostou A. Quality of life improves significantly after real-world oral immunotherapy for children with peanut allergy. Ann Allergy Asthma Immunol. (2020) 125(2):196–201.e1. 10.1016/j.anai.2020.03.028 32278075

[14] Blumchen K Trendelenburg V Ahrens F Gruebl A Hamelmann E Hansen G. Efficacy, safety, and quality of life in a multicenter, randomized, placebo-controlled trial of low-dose peanut oral immunotherapy in children with peanut allergy. J Allergy Clin Immunol Pract. (2019) 7(2):479–91.e10. 10.1016/j.jaip.2018.10.048 30423449

[15] Nagakura K Yanagida N Sato S Nishino M Asaumi T Ogura K. Low-dose oral immunotherapy for children with anaphylactic peanut allergy in Japan. Pediatr Allergy Immunol. (2018) 29(5):512–8. 10.1111/pai.12898 29603410

[16] Martorell A Alonso E Echeverría L Escudero C García-Rodríguez R Blasco C. Oral immunotherapy for food allergy: a Spanish guideline. Egg and milk immunotherapy Spanish guide (ITEMS GUIDE). part 2: maintenance phase of cow milk (CM) and egg oral immunotherapy (OIT), special treatment dosing schedules. Models of dosing schedules of OIT with CM and EGG. Allergol Immunopathol (Madr). (2017) 45(5):508–18. 10.1016/j.aller.2017.05.002 28676231

[17] Hemmings O Du Toit G Radulovic S Lack G Santos AF. Ara h 2 is the dominant peanut allergen despite similarities with ara h 6. J Allergy Clin Immunol. (2020) 146(3):621–30.e5. 10.1016/j.jaci.2020.03.026 32298698 PMC7482438

[18] Ma D Zhu R. Low-dose oral immunotherapy in immunoglobulin E-mediated food allergies. Front Immunol. (2024) 15:1321863. 10.3389/fimmu.2024.1321863 38361918 PMC10867954

[19] Tamas TP Ciurariu E. Allergen immunotherapy: pitfalls, perks and unexpected allies. Int J Mol Sci. (2025) 26(8):3535. 10.3390/ijms26083535 40332034 PMC12027104

[20] Shi L Xiong P Yang M Ardicli O Schneider SR Funch AB. Role of IgG4 antibodies in human health and disease. Cells. (2025) 14(9):639. 10.3390/cells14090639 40358163 PMC12071442

[21] Layhadi JA Lalioti A Palmer E Van Zelm MC Wambre E Shamji MH. Mechanisms and predictive biomarkers of allergen immunotherapy in the clinic. J Allergy Clin Immunol Pract. (2024) 12(1):59–66. 10.1016/j.jaip.2023.11.027 37996041

[22] Bergmann MM Santos AF. Basophil activation test in the food allergy clinic: its current use and future applications. Expert Rev Clin Immunol. (2024) 20(11):1297–304. 10.1080/1744666X.2024.2336568 38591129

[23] Tsai M Mukai K Chinthrajah RS Nadeau KC Galli SJ. Sustained successful peanut oral immunotherapy associated with low basophil activation and peanut-specific IgE. J Allergy Clin Immunol. (2020) 145(3):885–96.e6. 10.1016/j.jaci.2019.10.038 31805311 PMC6957313

[24] Agyemang A Suprun M Suárez-Fariñas M Boina F Arif-Lusson R Grishin A. A novel approach to the basophil activation test for characterizing peanut allergic patients in the clinical setting. Allergy. (2021) 76(7):2257–9. 10.1111/all.14752 33506502

[25] Worm M Reese I Ballmer-Weber B Beyer K Bischoff SC Classen M. Guidelines on the management of IgE-mediated food allergies: s2k-guidelines of the German society for allergology and clinical immunology (DGAKI) in collaboration with the German medical association of allergologists (AeDA), the German professional association of pediatricians (BVKJ), the German allergy and asthma association (DAAB), German dermatological society (DDG), the German society for nutrition (DGE), the German society for gastroenterology, digestive and metabolic diseases (DGVS), the German society for oto-rhino-laryngology, head and neck surgery, the German society for pediatric and adolescent medicine (DGKJ), the German society for pediatric allergology and environmental medicine (GPA), the German society for pneumology (DGP), the German society for pediatric gastroenterology and nutrition (GPGE), German contact allergy group (DKG), the Austrian society for allergology and immunology (AE-GAI), German professional association of nutritional sciences (VDOE) and the association of the scientific medical societies Germany (AWMF). Allergo J Int. (2015) 24:256–93. 10.1007/s40629-015-0074-0 27069841 PMC4792347

[26] Turner PJ Ansotegui IJ Campbell DE Cardona V Carr S Custovic A. Updated grading system for systemic allergic reactions: joint statement of the world allergy organization anaphylaxis committee and allergen immunotherapy committee. World Allergy Organ J. (2024) 17(3):100876. 10.1016/j.waojou.2024.100876 38361745 PMC10867340

[27] Chinthrajah RS Jones SM Kim EH Sicherer SH Shreffler W Lanser BJ. Updating the CoFAR grading scale for systemic allergic reactions in food allergy. J Allergy Clin Immunol. (2022) 149(6):2166–70.e1. 10.1016/j.jaci.2021.12.789 35026206 PMC9177543

[28] Sonder SU Plassmeyer M Loizou D Alpan O. Towards standardizing basophil identification by flow cytometry. Front Allergy. (2023) 4:1133378. 10.3389/falgy.2023.1133378 36938328 PMC10020589

[29] Tang D Chen M Huang X Zhang G Zeng L Zhang G. SRplot: a free online platform for data visualization and graphing. PLoS One. (2023) 18(11):e0294236. 10.1371/journal.pone.0294236 37943830 PMC10635526

[30] Chowdhury EA Jadeja OC. The safety and efficacy of oral immunotherapy compared to epicutaneous immunotherapy in peanut allergen desensitisation amongst the paediatric cohort-a narrative review. Front Allergy. (2025) 6:1613237. 10.3389/falgy.2025.1613237 40463361 PMC12129782

[31] Suárez-Fariñas M Grishin A Arif-Lusson R Bourgoin P Matthews K Campbell DE. A streamlined strategy for basophil activation testing in a multicenter phase III clinical trial. J Allergy Clin Immunol Pract. (2024) 12(12):3383–92.e8. 10.1016/j.jaip.2024.09.007 39284563

[32] Shamji MH Kappen JH Akdis M Jensen-Jarolim E Knol EF Kleine-Tebbe J. Biomarkers for monitoring clinical efficacy of allergen immunotherapy for allergic rhinoconjunctivitis and allergic asthma: an EAACI position paper. Allergy. (2017) 72(8):1156–73. 10.1111/all.13138 28152201

[33] Pessina B Sertori C Barni S Catamerò F Liccioli G Paladini E. Peanut oral immunotherapy in children: an Italian single-center retrospective cohort study. Nutrients. (2026) 18(8):1252. 10.3390/nu18081252 42075065 PMC13118600

[34] Keswani T LaHood NA Marini-Rapoport O Karmakar B Andrieux L Reese B. Neutralizing IgG(4) antibodies are a biomarker of sustained efficacy after peanut oral immunotherapy. J Allergy Clin Immunol. (2024) 153(6):1611–20.e7. 10.1016/j.jaci.2024.02.017 38460677 PMC11894811

