# Supplementary material for: Longitudinal basophil activation testing for assessing response to low-dose initiation, slow-escalation peanut oral immunotherapy in patients with high Ara h 2-sIgE
Source: Front Allergy. 2026 Oct 7;7:1902025. doi: 10.3389/falgy.2026.1902025 (PMC13683690; doi:10.3389/falgy.2026.1902025)
Supplement: Supplementary file 1 [file Datasheet1.pdf]

Longitudinal basophil activation testing for assessing response to low-dose initiation, slow-escalation peanut oral immunotherapy in patients with high Ara h 2 sIgE

Jinguo Chen[1†], J. Joanna Yu[1†], Jing Yu[2], Lili Wan[1,2], Joseph Chiao[1], Athena Economides[2], Dingfen Han[3], H. Henry Li[1,2]

[1] Virant Diagnostics Laboratory, Wheaton, Maryland

[2] Institute for Asthma and Allergy (IAA), Chevy Chase, Maryland

[3] Department of Medicine, School of Medicine, Johns Hopkins University, Baltimore, Maryland

Supplements - 1

Methods

***Study design and population**:* Patients were screened among those who received peanut oral immunotherapy (OIT) at the Institute for Asthma and Allergy (IAA, Chevy Chase, Maryland) between September 2021 and June 2025. Participant flow and longitudinal assessments are illustrated in **Figure S1**.

Inclusion criteria were a history of clinical reactivity to peanut, a positive skin prick test (SPT; wheal ≥3 mm), peanut-specific IgE (sIgE) >0.35 kU/L, and at least two basophil activation test (BAT) assessments performed ≥6 months apart. Of 49 patients who initiated clinical peanut OIT at IAA, 32 met these criteria and were included in this biomarker analytic cohort study. Seventeen patients were excluded: 9 had incomplete OIT follow-up (they did not complete the first 18 visits. 2 remained in active OIT but had been treated for less than 6 months and had not yet reached SCD ≥300 mg at the time of data cutoff, 2 switched to another therapy because of uncontrolled asthma, 5 were lost to follow-up and counted as nonattainment); importantly, no patient discontinued OIT because of treatment-related adverse reactions. The remaining 8 were excluded because they had fewer than two BAT assessments performed ≥6 months apart, BAT testing at only a single allergen concentration, or no BAT assessment because of concurrent cow's milk allergy or other personal reasons.

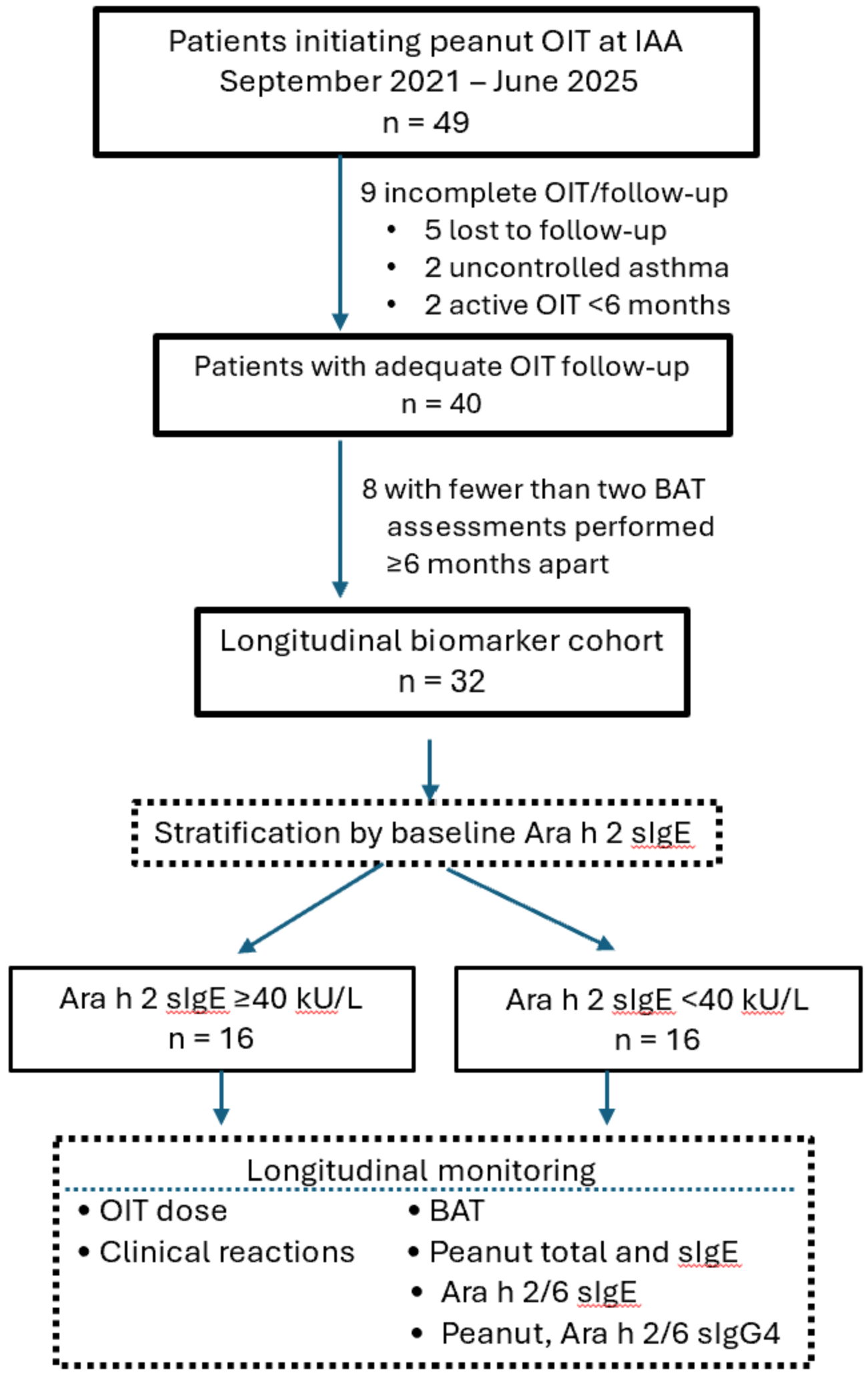

FIGURE S1. Study participant flow diagram and longitudinal monitoring. SPT: skin prick test; BAT: basophil activation test; OIT: oral immunotherapy

Peanut formulation and OIT Protocol*:* The peanut oral immunotherapy (OIT) protocol used in this study is briefly described below.

All patients underwent peanut OIT according to the clinical protocol used at IAA. The peanut protein content of the in-house prepared products was determined directly by laboratory analysis (26.5 mg and 212.1 mg of peanut protein per gram of MNB and PB, respectively). All administered doses used in the analysis, including doses <300 mg, were expressed as milligrams of peanut protein rather than total food-product protein. For commercially available products, total protein content was derived from manufacturer

nutrition labels as compiled in the USDA database. Although the values reflect the total protein content of Reese's Pieces, non-peanut proteins account for no more than 2% of the total, according to the product label. Therefore, the labeled total protein content was used as a practical estimate of peanut protein content for Reese's Pieces. The manufacturer-labeled total protein includes protein contributed by non-peanut ingredients for M&M's chocolate-coated peanut products and therefore may overestimate peanut protein content. Peanut protein content per M&M's piece was estimated according to Lazizi S, et al. (2022, *Front. Allergy* 3:974250. doi: 10.3389/falgy.2022.974250). The protein content of each formulation was standardized as described in **Table S1**.

TABLE S1. Formulas for Conversion of Peanut Products to Peanut Protein Dose

Product	Product amount administered	Peanut-protein contents	Source of peanut-content estimate
**MNB: Mixed nut butter**	1 tsp (~5 mL; 5.5 g butter)	26.5 mg/g	In-house (IAA)
**PB: Peanut butter**	1 tsp (~5 mL, 5.5 g butter)	212.1 mg/g	In-house (IAA)
**PN: Peanut**	1 whole peanut	200 mg/peanut	Literature
**M&M's –** Peanut Chocolate Candy	12 pieces (28 g)	250 mg/piece	Lazizi et al. (2022)
**Reese's Pieces -** Peanut Butter Candy	38 piece (30 g)	78.9 mg/piece	USDA Branded Food Data
**PB2: peanut butter powder**	2 tbsp (~30 mL; ~13 g powder)	3000 mg/tbsp	USDA Branded Food Data

IAA: Institute for Asthma and Allergy; USDA: U.S. Department of Agriculture, tsp: teaspoon

Peanut OIT consisted of two phases: an up-dosing phase and a maintenance phase. During the up-dosing phase, patients initially consumed a very small amount of a peanut-containing formulation prepared at IAA. Under physician supervision, patients were instructed to consume the prescribed dose daily at home. The dose was subsequently increased every 14–28 days, provided that the preceding dose was tolerated. Doses were increased stepwise over at least 17 increments (18 visits), with a target dose exceeding the 300-mg clinically meaningful threshold, as shown in **Table S2**. After crossing 300 mg/day, patients could either continue dose escalation to increase their tolerated daily

dose or transition to the maintenance phase, according to the clinical judgment of the treating physician and patient preference.

TABLE S2. Peanut OIT visits and dosages

**Visit**	**V1**	**V2**	**V3**	**V4**	**V5**	**V6**
**Dose (mg)**	0.1	0.3	0.4	0.7	1.5	2.2
**Visit**	**V7**	**V8**	**V9**	**V10**	**V11**	**V12**
**Dose (mg)**	4.6	9.3	18.5	37.0	74.1	111.1
**Visit**	**V13**	**V14**	**V15**	**V16**	**V17**	**V18**
**Dose (mg)**	148.2	182.2	222.2	250.0	291.5	364.4

Dose increases were undertaken only when the preceding dose had been tolerated for the prescribed period. If a patient experienced a mild adverse effect, graded according to the WAO and CoFAR criteria (Version 3.0), dose escalation could be deferred until the preceding dose was tolerated. Following a systemic allergic reaction, dosing was reduced to the most recent previously tolerated dose. A dose reduction was also considered when a patient missed OIT dosing for more than one week, with the subsequent dose determined by the treating physician based on the duration of the interruption and the patient's clinical status.

Once patients reached an individualized maintenance dose determined by the treating physician in consultation with the patient and family, they entered the maintenance phase. Patients continued to consume the established maintenance dose daily and returned to the physician's office approximately every 3 months. The planned maintenance duration was at least 3 years to sustain desensitization.

Basophil activation test (BAT): The BAT used in this study is an established laboratory-developed test (LDT) that underwent analytical validation before clinical implementation and is routinely performed in our CLIA-certified and CAP-accredited clinical laboratory. The original validation included accuracy/method comparability, intra-run repeatability, inter-run reproducibility, specimen stability, analytical specificity, and analytical sensitivity. Following implementation, assay performance has been monitored under the laboratory's ongoing quality-management program, including

positive- and negative-control monitoring, instrument performance monitoring, reagent lot verification, personnel competency assessment, and alternative proficiency assessment (APA).

Core analytical validation metrics are summarized in **Tables S3 and S4**. The clinical BAT positivity criterion was defined as ≥5% $CD63^+$ basophils based on published approaches and verified during laboratory validation.

Reagent lots are verified against predefined laboratory acceptance criteria before release for clinical testing. Lots that do not meet established criteria are not introduced into clinical use. Recent data from 11 lot-to-lot checks of the antibody master-mix are summarized in **Table S5**. The maximum observed lot-verification CV was 28.3%, within the predefined acceptance criterion of ≤30%.

To assess whether calendar-time assay drift could have contributed to longitudinal changes in BAT responses, positive-control responses were evaluated according to specimen processing date. The electronic QC dataset included 1,340 BAT specimens processed from January 2024 through July 2026. When summarized by calendar quarter, neither anti-IgE (p=0.651) nor fMLP (p=0.955) responses showed a significant temporal trend. These findings provide no evidence of progressive loss of positive-control responsiveness during the period evaluated and argue against calendar-time assay drift as an explanation for the observed reduction in peanut-induced BAT responses.

TABLE S3. Precision assessments based on %CD63+ basophil run in triplicates

Precision assessment	Sample	Control	Mean %CD63+	SD	CV (%)	Qualitative concordance
**Inter-run**	A22-0802	Anti-IgE	3.53	0.59	16.6	100%
	A23-0002	Anti-IgE	13.70	2.12	15.4	100%
	A22-0831	Anti-IgE	9.30	0.79	8.5	100%
	A22-0802	fMLP	59.87	3.67	6.1	100%
	A23-0002	fMLP	43.20	6.85	15.9	100%
	A22-0831	fMLP	75.97	3.54	4.7	100%
	A22-0802	Peanut*	8.50	1.47	17.3	100%
	A23-0002	Peanut	55.03	6.23	11.3	100%
	A22-0831	Peanut	1.93	0.35	18.2	100%
	A22-0802	CD45/CD123/CD193**	0.45	0.06	13.5	N/A
	A23-0002	CD45/CD123/CD193	0.76	0.12	15.9	N/A
	A22-0831	CD45/CD123/CD193	0.66	0.02	2.6	N/A
**Intra-run**	A22-0796	Anti-IgE	4.03	0.68	16.9	100%
	A22-0845	Anti-IgE	23.00	4.42	19.2	100%
	A22-0859	Anti-IgE	38.63	5.48	14.2	100%
	A22-0796	fMLP	49.17	4.93	10.0	100%
	A22-0845	fMLP	28.73	3.20	11.1	100%
	A22-0859	fMLP	46.57	1.81	3.9	100%
	A22-0796	Peanut	17.50	3.03	17.3	100%
	A22-0845	Peanut	0.67	0.13	19.4	100%
	A22-0859	Peanut	67.07	3.19	4.8	100%
	A22-0796	CD45/CD123/CD193	0.57	0.01	2.0	N/A
	A22-0845	CD45/CD123/CD193	0.27	0.04	15.6	N/A
	A22-0859	CD45/CD123/CD193	0.52	0.02	4.0	N/A

* peanut at 10 µg/mL; ** CD45/CD123/CD193 : % Basophils in white blood cells

TABLE S4. Other core analytical validation performance for BAT

Validation parameter	Study design	Key results	Overall interpretation
**Accuracy / method comparability**	20 samples tested on Attune V4 (IAA) and Attune V6 (Virant Diagnostics)	Pearson correlation p < 0.01; trends for both CD63 and CD203c were concordant; 100% concordance for reportable (+/−) results	Excellent inter-instrument comparability
**Post-collection stability**	Whole blood stored at 18–25°C for 0, 1, and 2 days before BAT testing	Significant correlation between Day 0 and Days 1/2 (p < 0.01); activated basophil percentage (%CD63+) generally CV <20% versus Day 0; positive-control induction remained >90% for %CD63+	Acceptable sample stability: stable for approximately 24 h at 18–25°C before BAT processing

TABLE S5. Summary of Recent 11 lot-to-lot check data for antibody master-mix

Parameter	Summary
Total lot-to-lot checks	11
Period covered	Jan 2023–Apr 2026
CD45/IgE identification	Concordant/pass in 10/11 initial checks; one required repeat testing and passed on repeat
CD45/CD123/CD193 identification	11/11 pass
Anti-IgE %CD63	Generally comparable; CV range 5.8–26.3%
fMLP %CD63	Generally comparable; CV range 0.1–25.8%
Overall assessment	Consistent lot-to-lot performance with no documented lot failure (CV<30%)
