# Supplementary material for: Longitudinal basophil activation testing for assessing response to low-dose initiation, slow-escalation peanut oral immunotherapy in patients with high Ara h 2-sIgE
Source: Front Allergy. 2026 Oct 7;7:1902025. doi: 10.3389/falgy.2026.1902025 (PMC13683690; doi:10.3389/falgy.2026.1902025)
Supplement: Supplementary file 2 [file Datasheet2.pdf]

Supplements - 2

FIGURE S2. Baseline peanut sIgE showed positive descriptive associations with Ara h 2- and Ara h 6-specific IgE. (**A**) Peanut sIgE vs. Ara h 2 sIgE; (**B)** Peanut sIgE vs. Ara h 6 sIgE. Because several measurements exceeded the assay upper limit, these associations are presented descriptively. These descriptive analyses illustrate concordant baseline sensitization patterns between whole-peanut and component-specific IgE.

FIGURE S2.

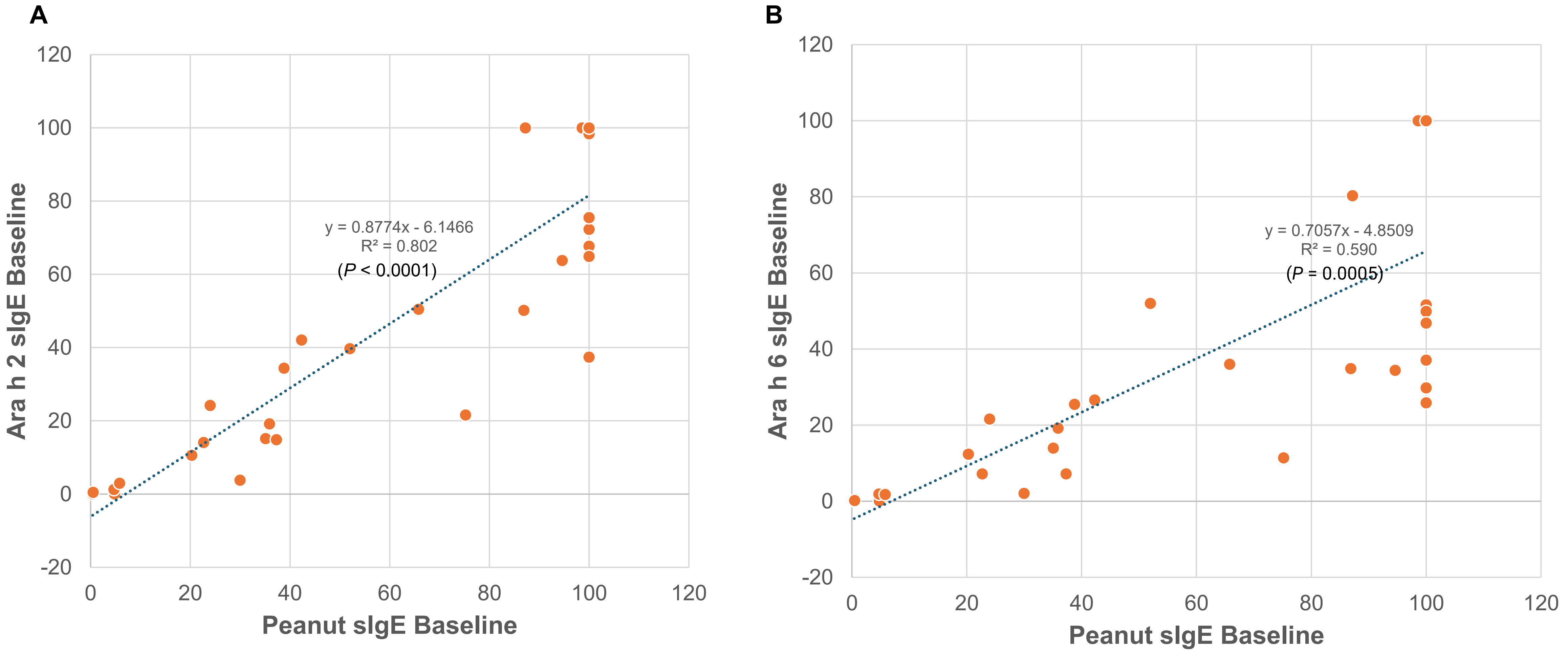
A
B
Ara h 2 sIgE Baseline
Peanut sIgE Baseline
y = 0.8774x - 6.1466
R² = 0.802
(P < 0.0001)
Ara h 6 sIgE Baseline
y = 0.7057x - 4.8509
R² = 0.590
(P = 0.0005)

FIGURE S3. Inter-individual variability in basophil activation patterns

Representative basophil activation test (BAT) dose–response curves from individual participants demonstrated heterogeneous response patterns, including bell-shaped and plateau profiles across serial peanut concentrations. This variability supports the use of integrated metrics, such as area under the curve (AUC), for analysis.

FIGURE S3:

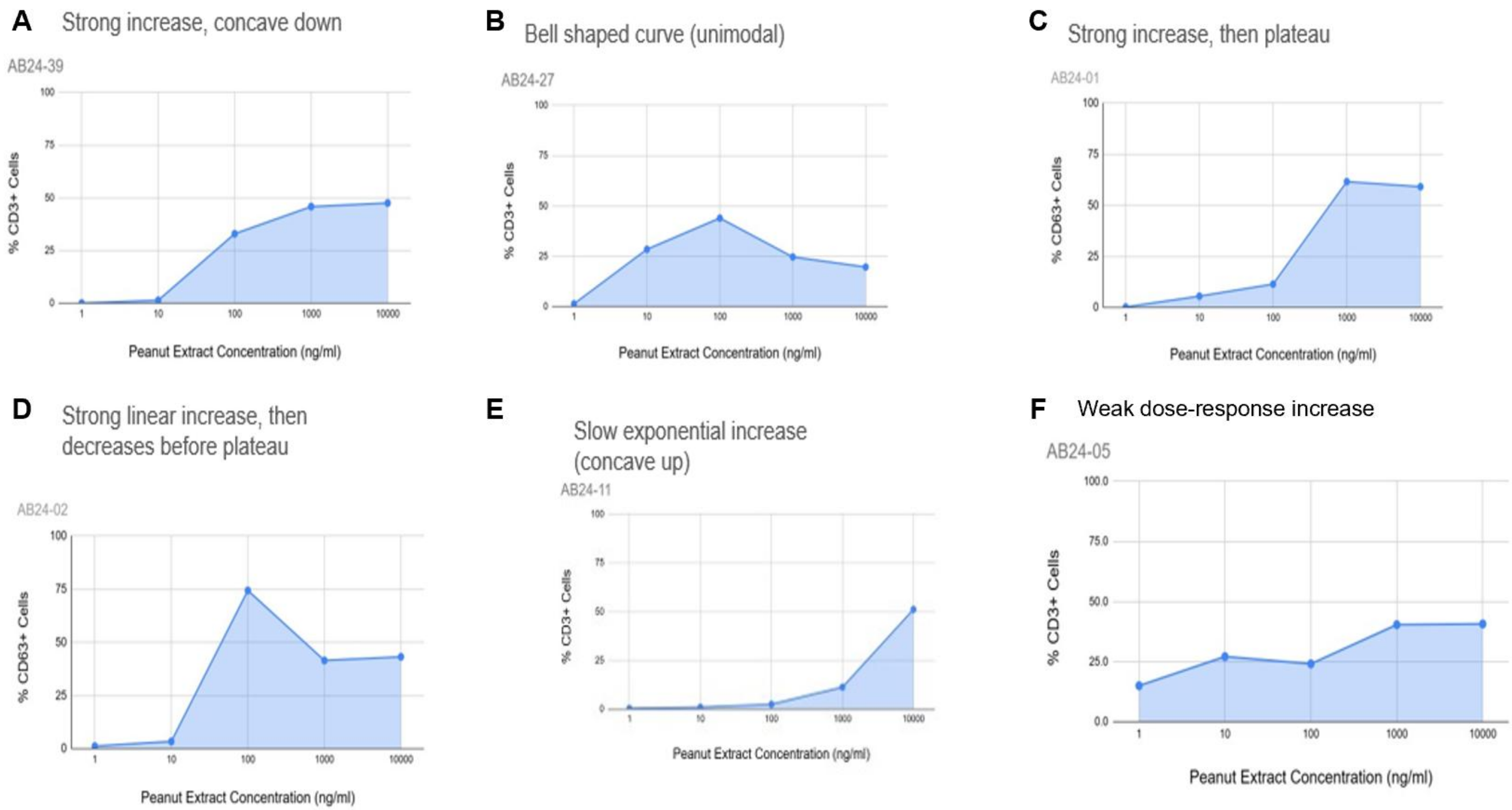
A
Strong increase, concave down
AB24-39
% CD3+ Cells
Peanut Extract Concentration (ng/ml)
B
Bell shaped curve (unimodal)
AB24-27
% CD3+ Cells
Peanut Extract Concentration (ng/ml)
C
Strong increase, then plateau
AB24-01
% CD63+ Cells
Peanut Extract Concentration (ng/ml)
D
Strong linear increase, then decreases before plateau
AB24-02
% CD63+ Cells
Peanut Extract Concentration (ng/ml)
E
Slow exponential increase (concave up)
AB24-11
% CD3+ Cells
Peanut Extract Concentration (ng/ml)
F
Weak dose-response increase
AB24-05
% CD3+ Cells
Peanut Extract Concentration (ng/ml)
1
10
100
1000
10000

FIGURE S4. Descriptive relationship between BAT AUC and maximum dose response. Scatter plot showing the descriptive relationship between basophil activation test (BAT) area under the curve (AUC) and maximum dose response (MDR, % $CD63^{+}$ basophils). AUC and MDR showed close concordance across BAT measurements, supporting use of AUC as an integrated summary of the dose-response curve.

FIGURE S4:

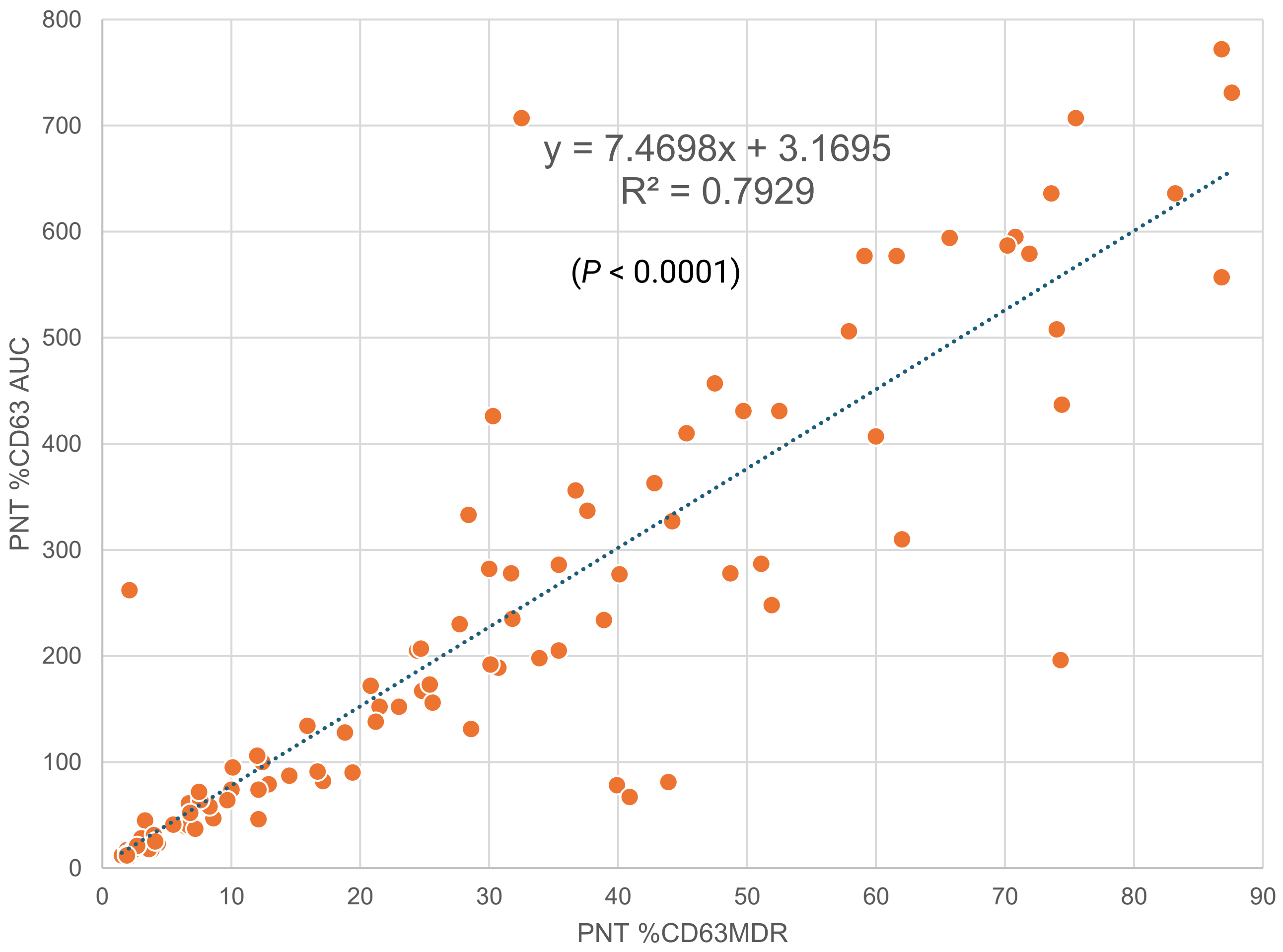

y = 7.4698x + 3.1695
R² = 0.7929
(P < 0.0001)
800
700
600
500
400
300
200
100
0
0
10
20
30
40
50
60
70
80
90
PNT %CD63 AUC
PNT %CD63MDR

FIGURE S5. Lack of predictive value of baseline biomarkers for OIT outcomes

(A) No significant correlation between baseline peanut sIgE and basophil activation test (BAT) area under the curve (AUC); (B) Baseline BAT AUC did not correlate with desensitization outcome (maximum SCD achieved); (C) Exploratory dichotomous cutoff analysis demonstrated no clinically useful baseline BAT threshold. These findings indicate limited utility of baseline biomarkers for predicting treatment outcomes. Panels A and B, n=25; panel C, n=25.

FIGURE S5.

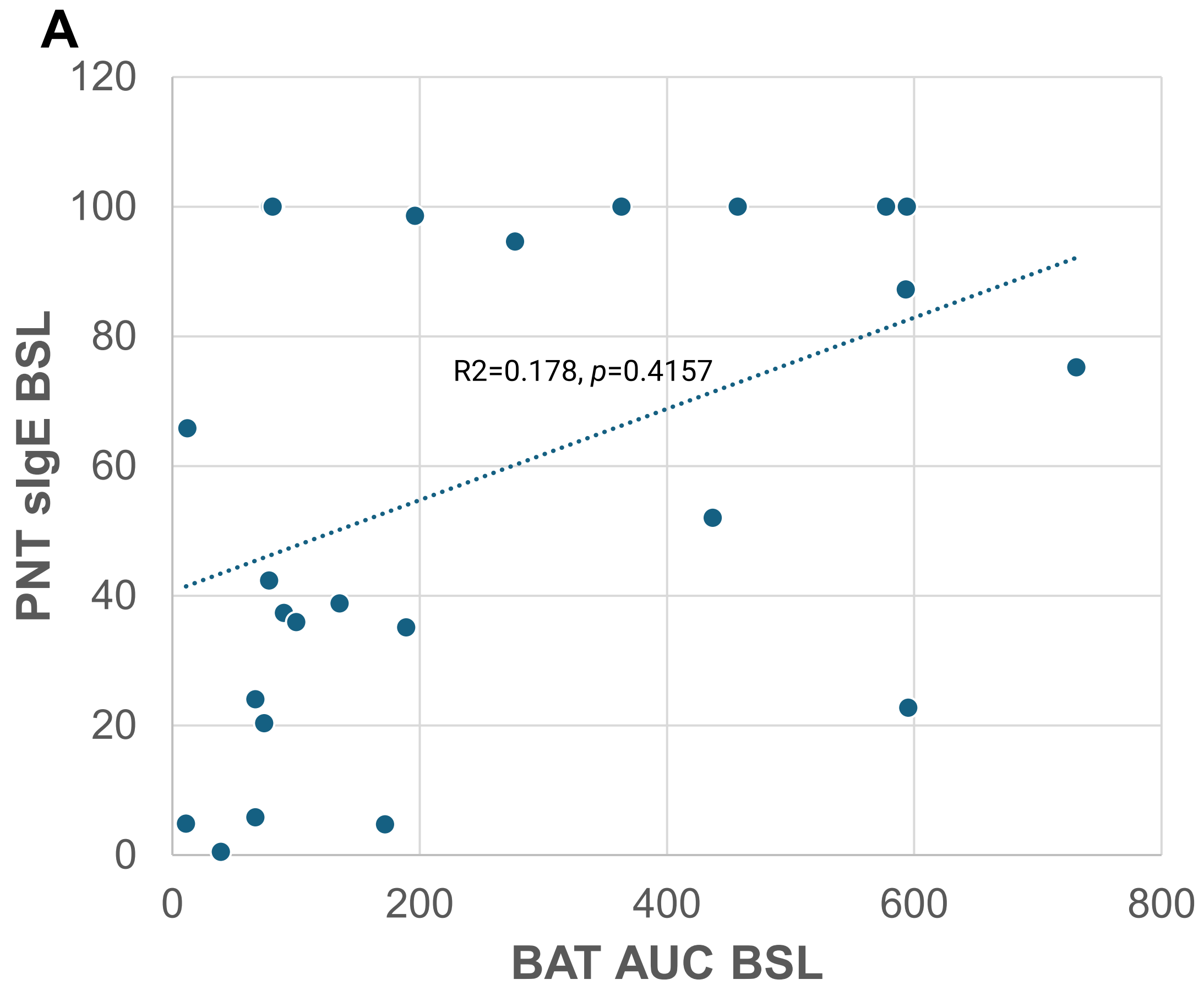

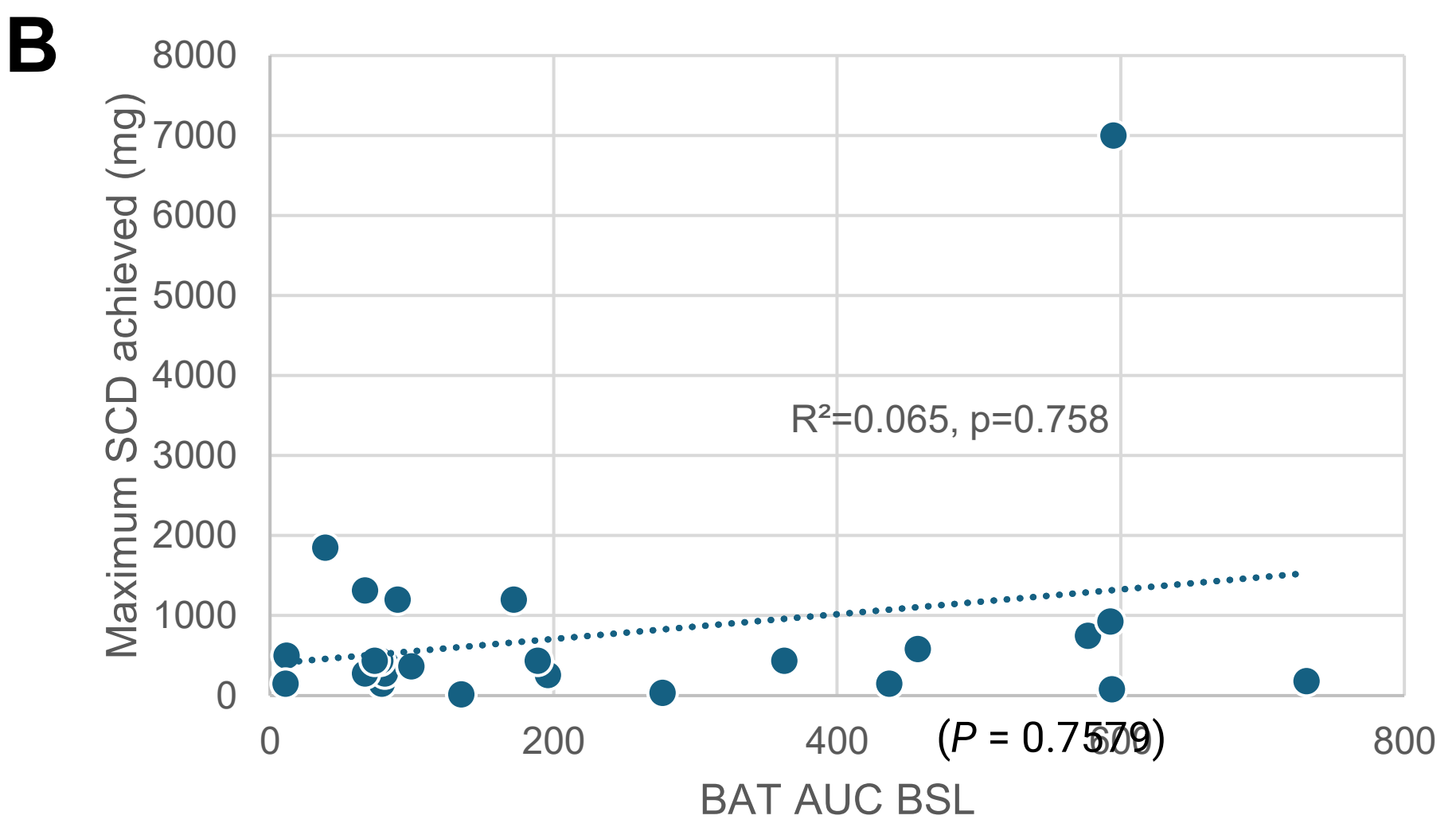

C

BAT Baseline (p=0.51)	SCD≥300mg	SCD<300mg
AUC <100	8	4
AUC≥100	7	6

FIGURE S6. Lack of association between baseline BAT and subsequent IgG4 response

Peanut-specific IgG4 did not differ significantly between participants with baseline BAT AUC <100 (n=12) and ≥100 (n=11; p=0.71). This finding suggests that early effector-cell responsiveness does not predict later humoral immune modulation in this small exploratory analysis.

FIGURE S6.

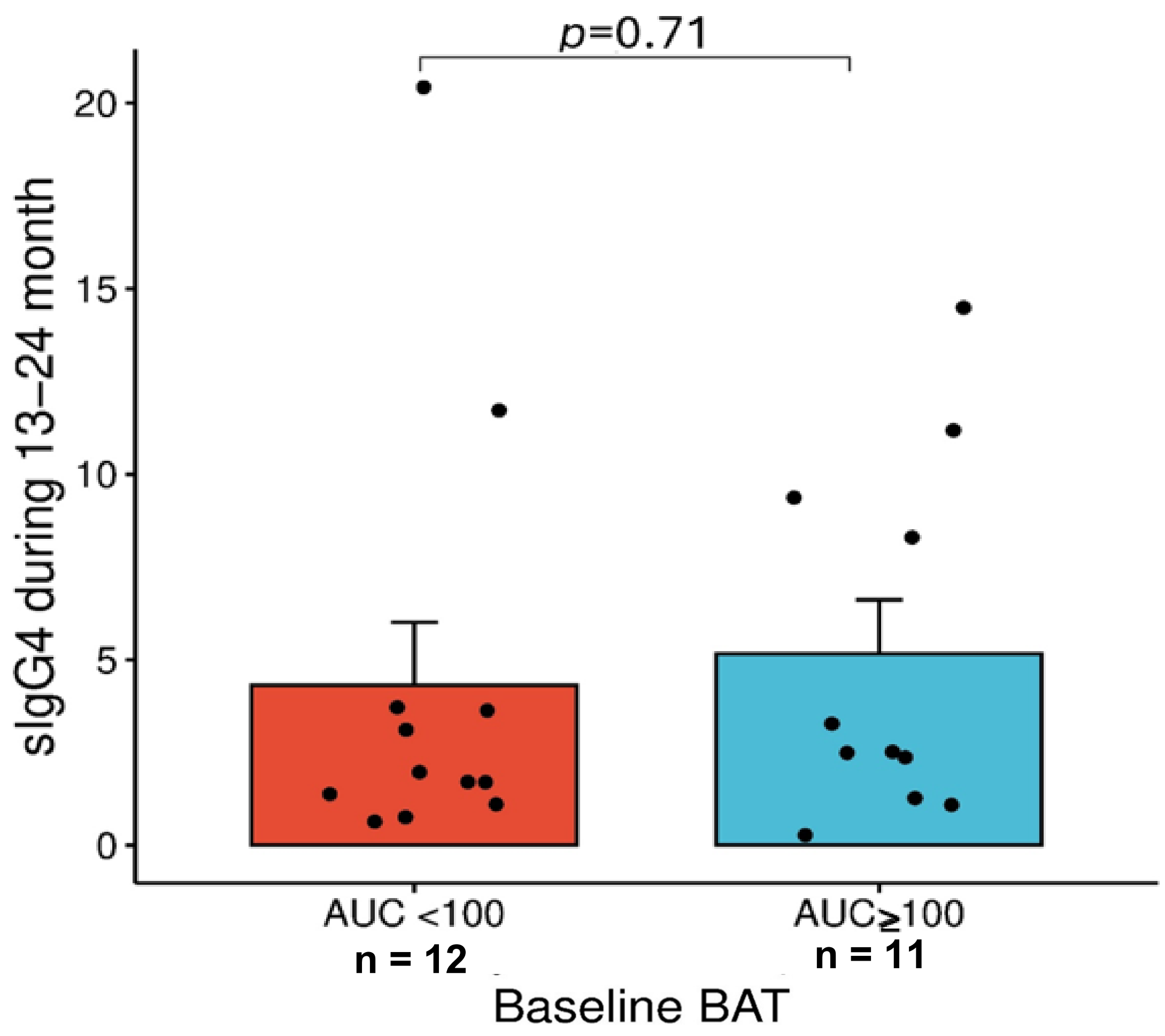

p=0.71
20
15
10
5
0
sIgG4 during 13–24 month
AUC <100
n = 12
AUC≥100
n = 11
Baseline BAT
